# Nrf2 and Parkin-Hsc70 regulate the expression and protein stability of p62/SQSTM1 under hypoxia

**DOI:** 10.1038/s41598-022-25784-0

**Published:** 2022-12-08

**Authors:** Ferbian Milas Siswanto, Yumi Mitsuoka, Misato Nakamura, Ami Oguro, Susumu Imaoka

**Affiliations:** 1 Department of Biomedical Chemistry, School of Biological and Environmental Sciences, Kwansei Gakuin University, Sanda, Japan; 2 Program of Biomedical Science, Graduate School of Integrated Sciences for Life, Hiroshima University, Hiroshima, Japan

**Keywords:** Stress signalling, Transcription factors, Ubiquitylated proteins

## Abstract

Solid tumors often contain regions with very low oxygen concentrations or hypoxia resulting from altered metabolism, uncontrolled proliferation, and abnormal tumor blood vessels. Hypoxia leads to resistance to both radio- and chemotherapy and a predisposition to tumor metastases. Under hypoxia, sequestosome 1 (SQSTM1/p62), a multifunctional stress-inducible protein involved in various cellular processes, such as autophagy, is down-regulated. The hypoxic depletion of p62 is mediated by autophagic degradation. We herein demonstrated that hypoxia down-regulated p62 in the hepatoma cell line Hep3B at the transcriptional and post-translational levels. At the transcriptional level, hypoxia down-regulated *p62* mRNA by inhibiting nuclear factor erythroid 2-related factor 2 (Nrf2). The overexpression of Nrf2 and knockdown of Siah2, a negative regulator of Nrf2 under hypoxia, diminished the effects of hypoxia on *p62* mRNA. At the post-translational level, the proteasome inhibitor MG132, but not the lysosomal inhibitors ammonium chloride and bafilomycin, prevented the hypoxic depletion of p62, suggesting the involvement of the proteasome pathway. Under hypoxia, the expression of the E3 ubiquitin ligase Parkin was up-regulated in a hypoxia-inducible factor 1α-dependent manner. Parkin ubiquitinated p62 and led to its proteasomal degradation, ensuring low levels of p62 under hypoxia. We demonstrated that the effects of Parkin on p62 required heat shock cognate 71 kDa protein (Hsc70). We also showed that the overexpression of Nrf2 and knockdown of Parkin or Hsc70 induced the accumulation of p62 and reduced the viability of cells under hypoxia. We concluded that a decrease in p62, which involves regulation at the transcriptional and post-translational levels, is critical for cell survival under hypoxia. The present results show the potential of targeting Nrf2/Parkin-Hsc70-p62 as a novel strategy to eradicate hypoxic solid tumors.

## Introduction

All metazoans require a sufficient supply of oxygen (O_2_), an essential substrate in various metabolic processes, bioenergetics, and cellular signaling. O_2_ deprivation or hypoxia (0.1–1% O_2_) is a fundamental feature of physiological and pathophysiological conditions, such as respiratory and circulatory disorders as well as cancer. In solid tumors, rapidly proliferating cancer cells cause tumor mass outgrowth and a shortage of blood supply, resulting in the formation of a hypoxic region^1^. Hypoxia plays a significant role in tumor development, contributes to therapeutic resistance, and is a critical microenvironmental factor promoting metastatic progression^2^, thereby serving as a prognostic factor as well as a compelling therapeutic target^3,4^. Central to the molecular and cellular responses to hypoxia, hypoxia-inducible factors (HIFs) sense and orchestrate various metabolic adaptation pathways and the transcriptional cascades of stress factors regulating cell death or survival^5^. This adaptation includes alterations in metabolism and nutrient acquisition^6^. Due to its central role in hypoxia responses, HIF and its target genes have emerged as promising therapeutic targets for solid tumor therapy. However, HIF inhibitors have not yet been approved for the treatment of cancer patients due to limited selectivity, specificity, and therapeutic efficacy or safety^7,8^. Therefore, the identification of new pathway(s) in hypoxic cell survival and the development of agents that selectively target components of this pathway may provide more effective approaches for solid tumor treatments.

Macroautophagy (hereafter referred to as autophagy), a key process in organelle turnover and nutrient management, is enhanced in response to hypoxia as part of the survival process^9–11^. Autophagy is a highly conserved catabolic process of intracellular molecules and organelles via lysosome-dependent degradation that maintains homeostatic cellular physiology. It is activated in hypoxia to ensure healthy cell function and metabolism to postpone the onset of cell death. Autophagy is broadly divided into the non-selective and selective autophagy of specific proteins or organelles and is activated by stresses, such as starvation, pathogen infection, oxidative stress, and hypoxia^12,13^. The autophagy receptor sequestosome 1 (SQSTM1/p62, hereafter called p62) is a multifunctional stress-inducible protein that is involved in various cellular processes by serving as a scaffold or as an adapter protein that facilitates protein quality control. The intracellular level of p62 is regulated by the balance between transcriptional regulation and post-translational degradation^14^. Several transcription factors have been found to regulate the basal and inducible expression of *p62*, including activator protein 1^15^, nuclear factor kappa B^16^, c-Jun^17^, and nuclear factor erythroid 2-related factor 2 (Nrf2)^18^. The post-translational regulation of p62 includes both autophagic degradation^19,20^ and proteasomal degradation pathways orchestrated by E3 ubiquitin ligases, such as tripartite motif-containing protein 21 (TRIM21)^21^ and Parkin^22^. The intracellular level of p62 regulated by autophagy-dependent or -independent mechanisms plays a decisive role in carcinogenesis^23^.

Under hypoxia, p62 protein expression is down-regulated. This hypoxic suppression of p62 is essential for cell survival because it up-regulates Ras/ERK signaling and subsequently reduces energy and O_2_ consumption^19,24^. The induction of apoptosis by hypoxia via the extrinsic FADD/caspase-8 and intrinsic p53 pathways is well-documented^25–28^. Studies showed that p62 was crucial for the activity of caspase-8^29^ and p53^30^ to induce apoptosis; therefore, the hypoxic depletion of p62 may impair apoptosis and improve cell survival under hypoxia. Given the potential involvement of p62 in hypoxic cell survival, a comprehensive understanding of the molecular mechanisms governing the depletion of p62 under hypoxia is essential for providing more detailed information on the development of therapeutic strategies.

Studies using human cervical carcinoma (HeLa)^19^ and glioma (U87MG and T98)^31^ cell lines as well as tumor-initiating cell-enriched patient-derived colorectal cancer^32^ demonstrated that the clearance of the p62 protein under hypoxia was exclusively attributed to autophagic degradation. In contrast, a study using human neuroblastoma (SH-SY5Y) cells showed that p62 was degraded under hypoxia by the proteasome system^22^. Additionally, independent research on head and neck squamous cell carcinoma (HNSCC)^33^ and an in vivo study on rat hippocampal tissue^34^ demonstrated that the mRNA expression of *p62* was down-regulated by hypoxia. These contradictory findings suggest that p62 has both pleiotropic and tissue-specific regulatory mechanisms and roles. Since different cells respond to hypoxia differently in regards to cell survival pathways and chemotherapeutic resistance^27^, we herein used human hepatocellular carcinoma cells (Hep3B) to characterize the regulation of p62 under hypoxia in hepatocytes in more detail. We also demonstrated that decreases in p62 under hypoxia in Hep3B cells occurred at both the transcriptional and post-translational levels via proteasomal degradation, but not autophagic degradation. The inhibition of Nrf2 under hypoxia was responsible for the down-regulation of *p62* mRNA. Additionally, hypoxia induced the activation of the Parkin-Hsc70 complex and subsequently caused the proteasomal degradation of the p62 protein. The present results showed that the depletion of p62 was critical for cancer cell survival under hypoxia, thereby suggesting novel strategies for the treatment of hypoxic solid tumors by targeting the pathways responsible for the depletion of p62.

## Results

### Effects of hypoxia on *p62* mRNA and protein levels

**Figure 1:**
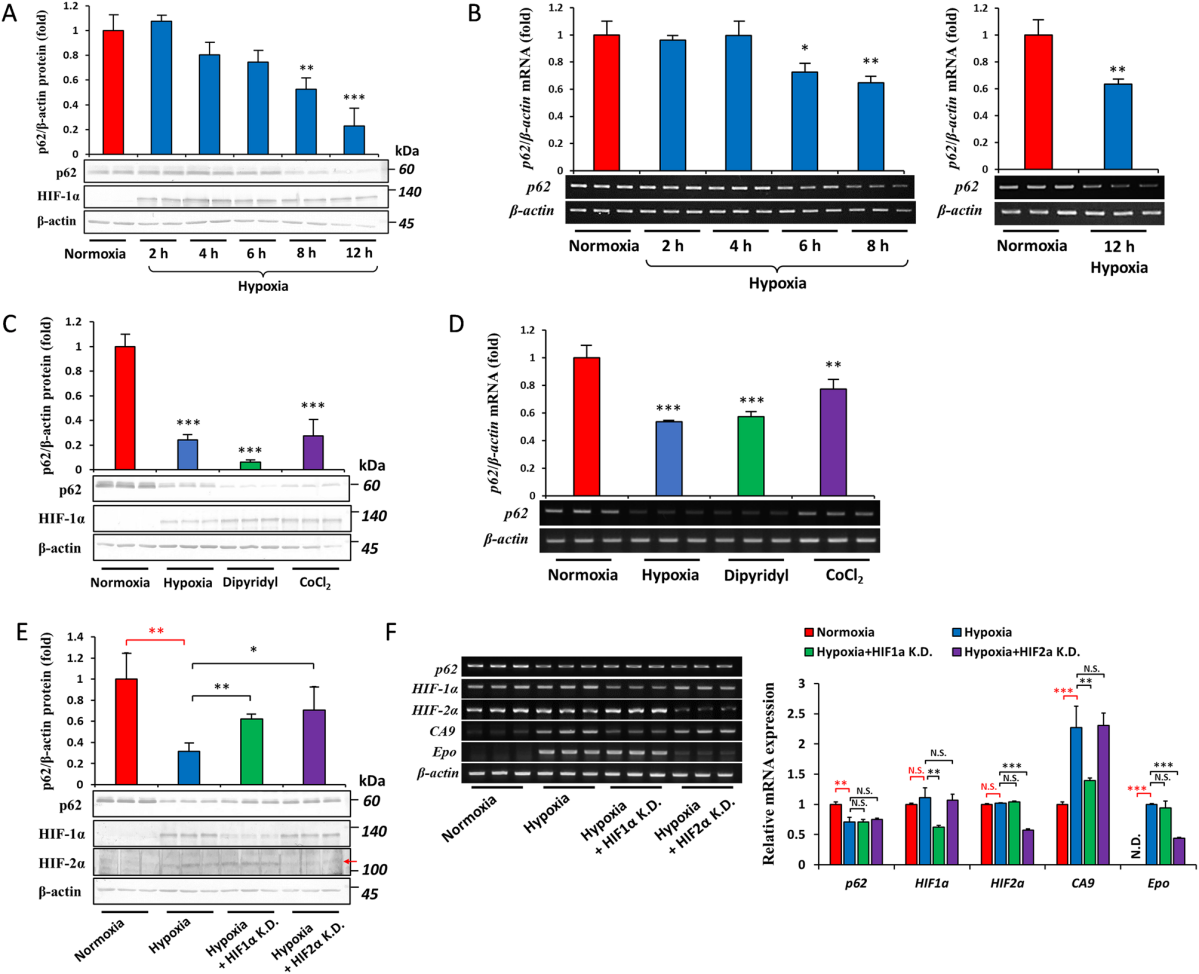
Hypoxia reduces *p62* mRNA and protein levels. (**A**) Hep3B cells were incubated under hypoxia (O_2_ = 1%) for 2, 4, 6, 8, and 12 h. The protein concentrations of p62, HIF-1α, and β-actin were examined by immunoblotting. (**B**) Hep3B cells were incubated under hypoxia (O_2_ = 1%) for the indicated period. The mRNA levels of *p62* were assessed by RT-PCR and quantified relative to *β-actin*. (**C** and **D**) Hep3B cells were incubated under hypoxia (O_2_ = 1%) or treated with 100 µM dipyridyl or 100 µM CoCl_2_ under normoxia for 12 h. The protein concentration (**C**) and mRNA abundance (**D**) of p62 were measured by immunoblotting and RT-PCR, respectively. (E and F) Hep3B cells were transfected with siRNA-Control, siRNA-HIF-1α (HIF-1α K.D.), or siRNA-HIF-2α (HIF-2α K.D.) and were incubated under hypoxia (O_2_ = 1%, 12 h). The protein concentration (**E**) and mRNA abundance (**F**) of p62, HIF-1α, HIF-2α, and β-actin were measured by immunoblotting and RT-PCR, respectively. Data are the mean ± S.D. of three replicates. N.S. not significant, *p < 0.05, **p < 0.01, ***p < 0.001 *versus* the indicated group, calculated with a one-way ANOVA with Dunnett’s post-hoc test (for figures **A**–**D**), a one-way ANOVA with Tukey’s post-hoc test (for figure **E**), or a multivariate one-way ANOVA with Tukey’s post-hoc test (for figure **F**).

Hep3B cells were incubated under hypoxia (O_2_ = 1%) for 2, 4, 6, 8, and 12 h, and the protein level of p62 was evaluated using immunoblotting. As shown in Fig. 1A, the cellular content of p62 decreased by approximately 40 and 80% after 8 and 12 h of hypoxia, respectively. To establish whether hypoxia reduced p62 by inhibiting its transcription, we examined the effects of hypoxia on the mRNA levels of *p62*. We found that *p62* mRNA levels were reduced by 6 to 12 h of hypoxia (Fig. 1B). Similarly, the hypoxia-mimetic agents, 2,2′-dipyridyl and cobalt chloride (CoCl_2_), which stabilize HIFs, also down-regulated the expression of *p62* mRNA (Fig. 1C) and protein (Fig. 1D) under normoxia. Since the roles of HIF-1α and HIF-2α in hypoxia-related tumorigenesis are well-documented^35,36^, we investigated whether these HIFs were involved in the hypoxic regulation of p62. Hep3B cells were transfected with siRNA against HIF-1α and HIF-2α followed by hypoxia for 12 h. We found that the knockdown of either isoform of HIF partially recovered p62 protein levels (Fig. 1E), suggesting that the depletion of p62 under hypoxia requires HIFs. The knockdown of HIF-1α or HIF-2α did not restore decreased *p62* mRNA levels (Fig. 1F), indicating that the role of HIFs in the hypoxic regulation of p62 occurred at the post-translational level.

### The role of Nrf2 on the hypoxic depletion of p62 at the transcriptional level

**Figure 2:**
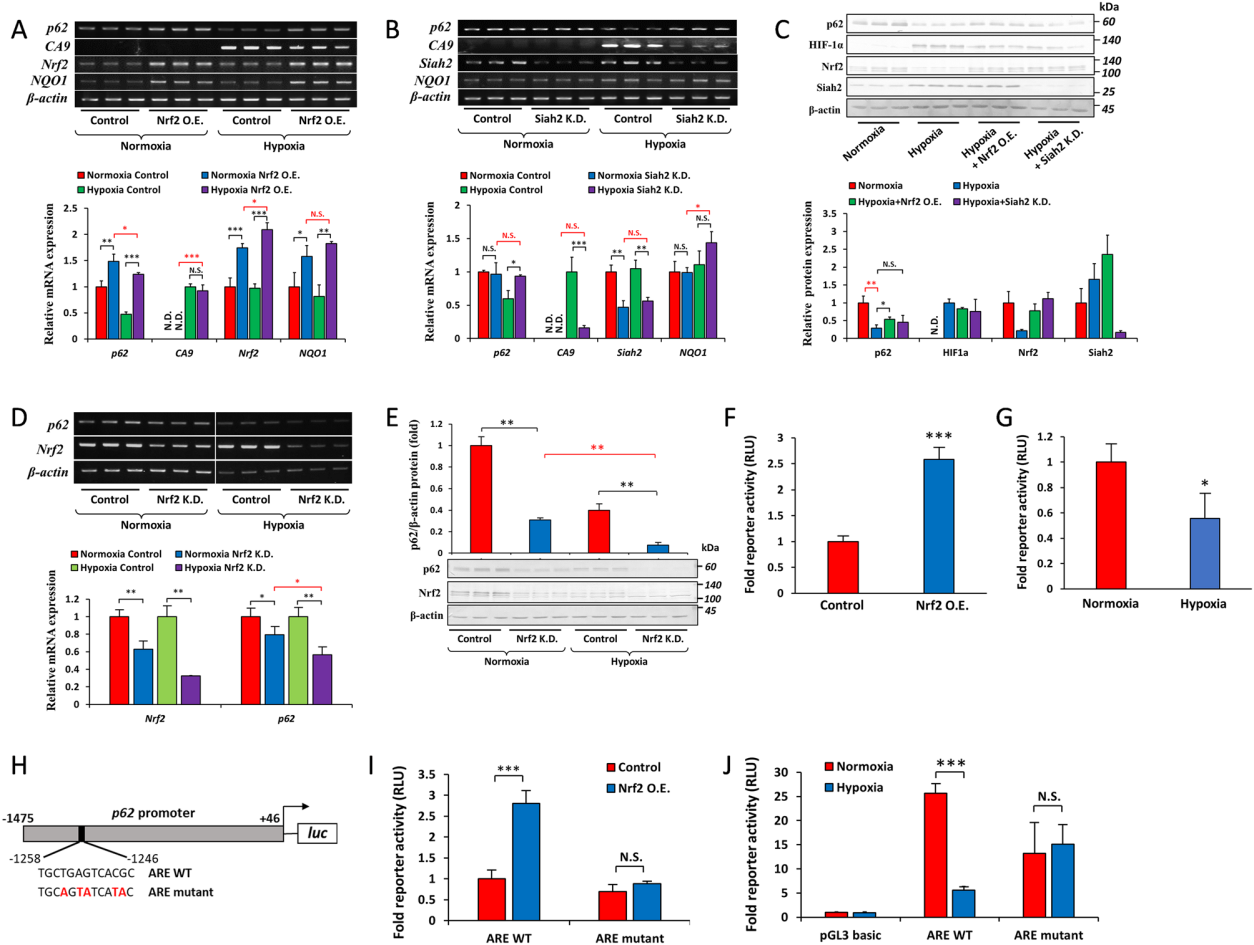
Depletion of *p62* mRNA under hypoxia requires the inhibition of Nrf2. (**A**) Hep3B cells were transfected with pcDNA-Mock (Control) or pcDNA-Nrf2 (Nrf2 O.E.), incubated under hypoxia (O_2_ = 1%, 12 h), and the mRNA levels of *p62*, *CA9*, *Nrf2*, *NQO1*, and *β-actin* were examined by RT-PCR. (**B**) Hep3B cells were transfected with shRNA-GFP (Control) or shRNA-Siah2 (Siah2 K.D.), incubated under hypoxia (O_2_ = 1%, 12 h), and the mRNA levels of *p62*, *CA9*, *Siah2*, *NQO1*, and *β-actin* were assessed by RT-PCR. (**C**) Hep3B cells were transfected with pcDNA-Nrf2 or shRNA-Siah2, incubated under hypoxia (O_2_ = 1%, 12 h), and the protein levels of p62, HIF-1α, Nrf2, Siah2, and β-actin were measured by immunoblotting. (**D** and **E**) Hep3B cells were transfected with siRNA-Control (Control) or siRNA-Nrf2 (Nrf2 K.D.), incubated under hypoxia (O_2_ = 1%, 12 h), and the mRNA (**D**) and protein (**E**) levels of p62, Nrf2, and β-actin were then measured by RT-PCR and immunoblotting, respectively. (**F**) The pGL3-containing upstream regions − 1475/ + 46 of *p62* were co-transfected with pRL-null and either pcDNA-Mock or pcDNA-Nrf2 into Hep3B cells. Luciferase activity was assessed 48 h after transfection. (**G**) The − 1475/ + 46 region of *p62* in the pGL3 vector was co-transfected with pRL-null, and cells were cultured under normoxia or hypoxia (O_2_ = 1%) for 12 h followed by the measurement of luciferase activity. (**H**) Wild-type ARE and its mutated construct within the *p62* promoter − 1475/ + 46. (**I**) The WT or mutant construct of the *p62* promoter was co-transfected with pRL-null and pcDNA-Mock or pcDNA-Nrf2 in Hep3B cells, and luciferase activity was measured. (J) The ARE WT or ARE mutant construct of the *p62* promoter in the pGL3 vector or pGL3-basic was co-transfected with pRL-null into Hep3B cells, and cells were cultured under normoxia or hypoxia (O_2_ = 1%) for 12 h followed by the measurement of luciferase activity. Data are the mean ± S.D. of triplicate experiments. N.S. not significant, *p < 0.05, **p < 0.01, ***p < 0.001 *versus* the indicated group, calculated with a multivariate one-way ANOVA with Tukey’s post-hoc test (for figures **A**, **B**, **C**, and **D**), a one-way ANOVA with Tukey’s post-hoc test (for figure **E**), a one-sample *t*-test (for figures **F** and **G**), or a one-sample *t*-test with Bonferroni’s correction (for figure **I** and **J**).

We investigated the mechanisms by which *p62* is transcriptionally regulated by hypoxia. Nrf2 has been reported to regulate the expression of *p62* as part of a positive feedback loop under conditions in which Nrf2 is activated^18^, and we previously showed that the Nrf2 pathway was suppressed under hypoxia by the E3 ligase Siah2^37^. Therefore, we examined whether Nrf2 was involved in the transcriptional regulation of *p62* under hypoxia. To achieve this, we overexpressed Nrf2 and knocked down Siah2 in Hep3B cells, followed by normoxia and hypoxia for 12 h. As expected, *p62* mRNA levels were markedly increased in cells overexpressing Nrf2 and cultured under both normoxia and hypoxia (Fig. 2A). By preventing the degradation of Nrf2 under hypoxia using the Siah2 knockdown strategy, the down-regulated expression of *p62* was also hampered (Fig. 2B). An examination of p62 further confirmed these results at the protein level. Decreased p62 protein levels under hypoxia were partially restored by the overexpression of Nrf2 and knockdown of Siah2 (Fig. 2C). The knockdown of Nrf2 under normoxia or hypoxia down-regulated the mRNA (Fig. 2D) and protein (Fig. 2E) expression of p62, suggesting that Nrf2 was also involved in the basal expression of *p62* under both conditions. To examine whether Nrf2 was involved in the reduced transactivation of the *p62* gene, the promoter activity of *p62* was assessed. The antioxidant response element (ARE) in the promoter region of *p62* was identified by Jain et al.^18^. Luciferase reporter plasmids (pGL3-basic) containing − 1475/ + 46 bp of the *p62* genomic region were transfected into Hep3B cells. Nrf2 overexpression (Fig. 2F) and hypoxia for 12 h (Fig. 2G) induced and reduced *p62* promoter activity, respectively. To verify the involvement of Nrf2 in the hypoxic inhibition of *p62* transactivation, the promoter containing the ARE mutant was constructed (Fig. 2H). We confirmed that the overexpression of Nrf2 did not induce the promoter activity of the ARE mutant-containing construct (Fig. 2I). As expected, hypoxia did not affect the reporter activity of ARE mutant reporter construct (Fig. 2J), highlighting the critical role of Nrf2 in the suppression of *p62* transactivation under hypoxia.

### Involvement of the proteasome pathway in the hypoxic clearance of p62

**Figure 3:**
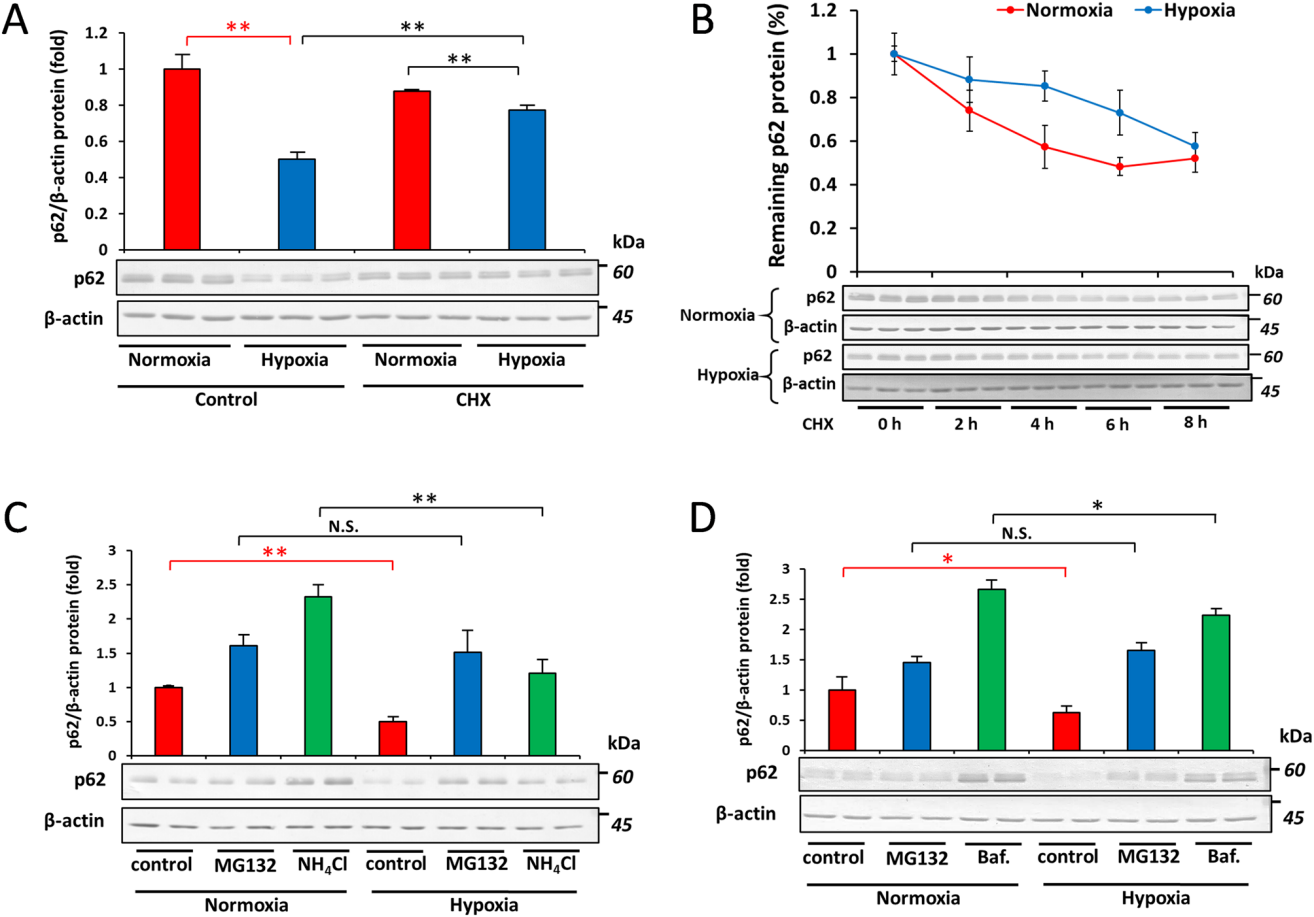
Involvement of the proteasome system in the degradation of p62 under hypoxia. (**A**) Hep3B cells were treated with vehicle (DMSO) or 10 μM cycloheximide and cultured under normoxia or hypoxia (O_2_ = 1%) for 12 h. The protein abundance of p62 was then examined by immunoblotting. (**B**) Hep3B cells were treated with 10 μM cycloheximide and cultured under normoxia or hypoxia (O_2_ = 1%) for the indicated period of time. p62 protein levels were then assessed by immunoblotting. (**C**) Hep3B cells were treated with vehicle (DMSO), 5 μM MG132, or 10 mM NH_4_Cl and cultured under normoxia or hypoxia (O_2_ = 1%) for 12 h followed by immunoblotting for the measurement of p62 protein levels. (**D**) Hep3B cells were treated with vehicle (DMSO), 5 μM MG132, or 10 nM bafilomycin and cultured under normoxia or hypoxia (O_2_ = 1%) for 12 h. p62 protein levels were quantified by immunoblotting. Data are presented as the mean ± S.D. of triplicate experiments. N.S. not significant, *p < 0.05, **p < 0.01 *versus* the indicated group, calculated with a one-way ANOVA with Tukey’s post-hoc test.

Since we provided evidence to show that the overexpression of Nrf2 did not fully recover the mRNA expression of *p62* and also that HIFs were involved in the post-transcriptional regulation of the p62 protein, we investigated whether hypoxia induced the degradation of the p62 protein in Hep3B, as was previously reported in HeLa and U87MG cells^19,31^. We treated Hep3B cells with the translation inhibitor cycloheximide (CHX) and found that hypoxia still reduced the amount of p62 (Fig. 3A). We then performed the CHX-chase assay to establish whether hypoxia accelerated the degradation of p62. We found that the half-life of p62 was shorter in cells cultured under hypoxia than in those cultured under normoxia (Fig. 3B). These results suggest that transcriptional modulation played a more dominant role in the suppression of p62 under hypoxia than its post-translational degradation. We then examined the mechanisms underlying the degradation of p62 under hypoxia. We compared the effects of the proteasomal inhibitor MG132 and the autophagic inhibitors, ammonium chloride (NH_4_Cl) and bafilomycin A1. The results obtained showed that hypoxia reduced p62 protein levels in the presence of NH_4_Cl and bafilomycin A1, but not MG132 (Fig. 3C and D), suggesting that p62 was regulated by a proteasomal-dependent pathway under hypoxia.

### The role of Parkin-Hsc70 in the hypoxic depletion of the p62 protein

**Figure 4:**
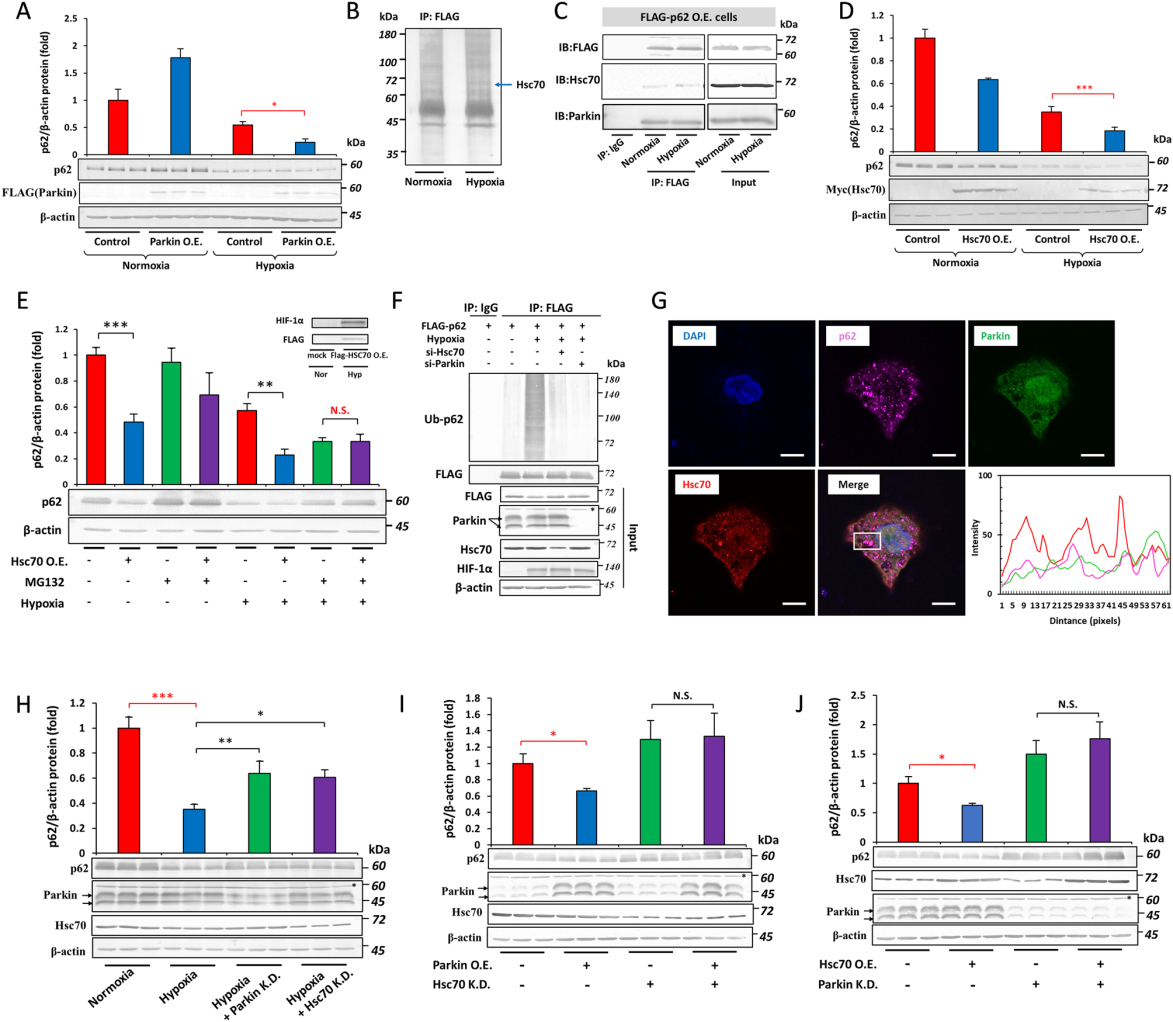
The Parkin-Hsc70 complex regulates the stability of the p62 protein under hypoxia. (**A**) Parkin in 3 × FLAG-pcDNA4 was transfected into Hep3B cells, cells were then incubated under normoxia or hypoxia (O_2_ = 1%) for 12 h, and p62 protein levels were examined by immunoblotting. (**B**) FLAG-Parkin was overexpressed in Hep3B cells, cultured under normoxia or hypoxia for 12 h, and immunoprecipitated with the anti-FLAG antibody. The band indicated by the arrow was analyzed by LC–MS. (**C**) Hep3B cells were transfected with the FLAG-p62, cell lysates were immunoprecipitated with an anti-FLAG antibody, and immunoprecipitated proteins were subjected to an immunoblotting analysis with anti-FLAG, anti-Hsc70, and anti-Parkin antibodies. (**D**) Hep3B cells were transfected with FLAG-Hsc70, incubated under normoxia or hypoxia for 12 h, and p62 protein levels were examined by immunoblotting. (**E**) FLAG-Hsc70 was transfected into Hep3B cells, incubated under normoxia or hypoxia for 12 h, treated with 5 µM MG132, and p62 protein levels were examined by immunoblotting. (**F**) FLAG-p62 was transfected with siRNA-Control, siRNA-Parkin, or siRNA-Hsc70, grown under normoxia or hypoxia for 12 h in the presence of 5 µM MG132, and cell lysates were immunoprecipitated using an anti-FLAG antibody, and subjected to immunoblotting. (**G**) Hep3B cells were transfected with pcDNA-Parkin-Venus and the cellular co-localization of p62 (magenta), Parkin (green), Hsc70 (red), and DAPI (blue) was examined by immunofluorescence, scale bar: 10 μm. The histogram shows the fluorescence intensity of channels along the white box in the merged image. (H) Hep3B cells were transfected with siRNA-Control, siRNA-Parkin (Parkin K.D.), or siRNA-Hsc70 (Hsc70 K.D.), incubated under normoxia or hypoxia for 12 h, and p62 protein levels were examined by immunoblotting. (**I**) pcDNA-Parkin was co-transfected with siRNA-Control or siRNA-Hsc70, incubated under hypoxia for 12 h, and subjected to immunoblotting to assess p62 protein levels. (**J**) pcDNA-Hsc70 was co-transfected with siRNA-Control or siRNA-Parkin, incubated under hypoxia for 12 h, and subjected to immunoblotting. Data are presented as the mean ± S.D. of triplicate experiments. N.S. not significant, *p < 0.05, ***p < 0.001 versus the indicated group, calculated with a one-way ANOVA with Tukey’s post-hoc test.

We attempted to identify which E3 ligase plays a role in the hypoxic degradation of p62. A previous study demonstrated that TRIM21^21^ and Parkin^22^ were potent regulators of p62 protein stability under normoxia. However, the overexpression of TRIM21 under normoxia and hypoxia did not affect p62 protein stability (data not shown). In contrast, the overexpression of Parkin reduced the intracellular abundance of p62 under hypoxia only (Fig. 4A). To gain further insights into the mechanisms by which Parkin regulates the hypoxic degradation of p62, we overexpressed FLAG-tagged Parkin in Hep3B cells and immunoprecipitated FLAG-Parkin from cells cultured under normoxia and hypoxia. A mass spectroscopic analysis was performed, and the protein band indicated by the arrow was identified as heat shock cognate 71 kDa protein (Hsc70) (Fig. 4B). The interactions of p62 with Parkin and Hsc70 were then examined by immunoprecipitation. Hep3B cells were overexpressed with FLAG-p62, and the analysis of immunoprecipitated lysates by immunoblotting showed that p62 interacted with endogenous Parkin and Hsc70 (Fig. 4C). We also assessed the role of Hsc70 in the regulation of p62. We transfected Hep3B cells with Myc-tagged Hsc70 under normoxia and hypoxia. The protein abundance of p62 was markedly reduced by the overexpression of Hsc70 under normoxia or hypoxia (Fig. 4D). Hsc70 was involved in the hypoxic proteasomal degradation of p62 because MG132 abolished the effects of the overexpression of Hsc70 on p62 under hypoxia (Fig. 4E). We then examined whether Parkin and Hsc70 ubiquitinated p62 under hypoxia. Under hypoxia, p62 was highly ubiquitinated, and the knockdown of Parkin or Hsc70 significantly decreased the ubiquitination of p62 (Fig. 4F).

To analyze the subcellular distribution of p62, Parkin, and Hsc70 in Hep3B cells and their potential to colocalize, we overexpressed Venus-tagged Parkin and its colocalization with p62 and Hsc70 was observed using immunofluorescence (Fig. 4G). A data analysis using the ImageJ RGB profiler plugin suggested that p62, Parkin, and Hsc70 colocalized to specific regions of the cytoplasm. Pearson’s coefficient indicated the modest co-localization of all proteins, with the strongest to weakest correlations in the following order: p62 and Hsc70 > Parkin and Hsc70 > p62 and Parkin (r = 0.711, 0.601, and 0.347, respectively). To further substantiate the role of Parkin and Hsc70 in the hypoxic suppression of p62, we transfected Hep3B cells with siRNA against Parkin and Hsc70 and cultured them under hypoxia. The depletion of p62 by hypoxia was blunted by the knockdown of Parkin and Hsc70 (Fig. 4H). These results prompted us to verify whether Parkin and Hsc70 work as a complex to regulate p62 stability under hypoxia. We demonstrated that Parkin overexpression in Hsc70 knockdown cells (Fig. 4I) and Hsc70 overexpression in Parkin knockdown cells (Fig. 4J) did not affect p62 protein levels. Collectively, these results showed that p62 was directly regulated by the Parkin-Hsc70 complex under hypoxia via the ubiquitin-dependent proteasome pathway.

### Effects of hypoxia on the expression of *Parkin*

**Figure 5:**
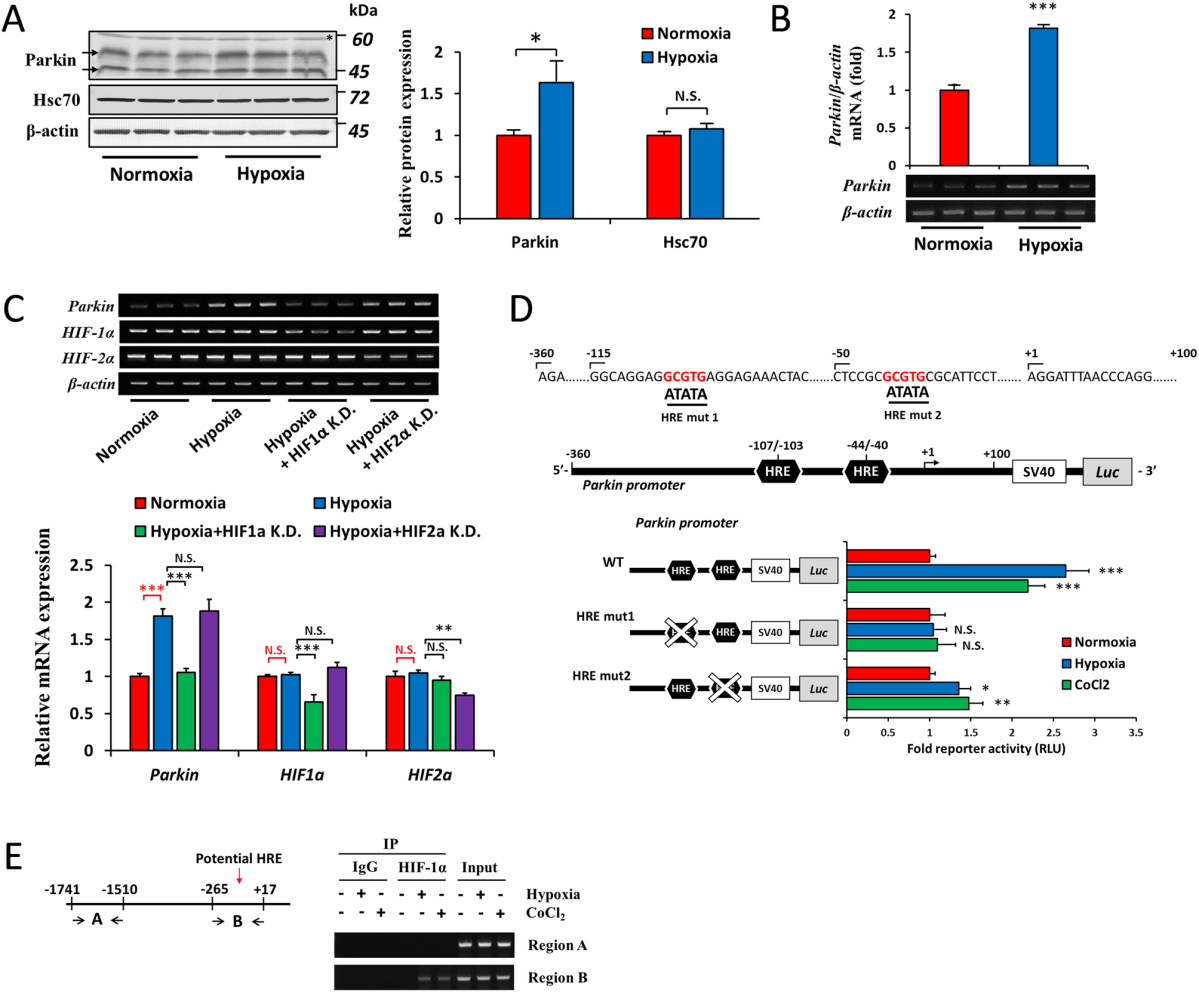
HIFs regulate the expression of *Parkin*. (A and B) Hep3B cells were cultured under normoxia or hypoxia (O_2_ = 1%) for 12 h, and the protein expression of Parkin and Hsc70 (**A**) and the mRNA level of Parkin (**B**) were assessed by immunoblotting and RT-PCR, respectively. (**C**) Hep3B cells were transfected with siRNA-Control, siRNA-HIF-1α (HIF-1α K.D.), or siRNA-HIF-2α (HIF-2α K.D.) and incubated under hypoxia (O_2_ = 1%, 12 h). The mRNA abundance of *Parkin*, *HIF-1α*, *HIF-2α*, and *β-actin* was measured by RT-PCR. (**D**) Wild-type hypoxia response elements (HREs) and their mutated constructs (HRE mut 1 and 2) within the *Parkin* promoter − 360/ + 100 (upper panel) were inserted into the pGL3 vector. These constructs were co-transfected with pRL-null into Hep3B cells and then cultured under normoxia or hypoxia (O_2_ = 1%) or treated with CoCl_2_ (100 µM, normoxia) for 12 h. Luciferase activity was then measured. (**E**) Genomic positions of *Parkin* regions that were selected for the ChIP assay (left panel). Hep3B cells were incubated under normoxia or hypoxia (O_2_ = 1%) or treated with CoCl_2_ (100 µM, normoxia) for 12 h, and the ChIP assay was performed with the anti-HIF-1α antibody or control rabbit IgG, with input chromatin as the positive control. After reverse cross-linking, DNA was amplified using the indicated primer sets (right panel). Data are the mean ± S.D. of triplicate experiments. N.S. not significant, *p < 0.05, **p < 0.01, ***p < 0.001 *versus* the indicated group, calculated with a one-sample *t*-test with Bonferroni’s correction (for figure A), a one-sample *t*-test (for figures **B**), or a multivariate one-way ANOVA with Tukey’s post-hoc test (for figures **C** and **D**).

Since we demonstrated that HIFs and Parkin-Hsc70 both regulated the post-translational regulation of p62 under hypoxia, we were interested in examining the crosstalk between these pathways. Previous studies reported that Parkin was activated by hypoxia and contributed to the induction of mitophagy in renal tubular and pulmonary artery smooth muscle cells^38,39^. In the present study, we found that hypoxia induced the expression of the Parkin protein (Fig. 5A) and mRNA (Fig. 5B) in Hep3B cells. Hsc70 levels were unaffected by the hypoxia treatment (Fig. 5A). We then investigated the roles of HIF-1α and HIF-2α in the induction of *Parkin* by hypoxia. While the knockdown of HIF-1α abrogated the effects of hypoxia on *Parkin* expression, the knockdown of HIF-2α did not (Fig. 5C), suggesting that only HIF-1α was involved in the induction of *Parkin*. To establish whether HIFs transactivated the *Parkin* gene, we used a reporter construct containing − 360/ + 100 base pairs of the human *Parkin* promoter fused to luciferase. Using the JASPAR database, we predicted two putative hypoxia response elements (HREs) within this region (Fig. 5D, upper panel). To verify the contribution of these HREs to the functional activity of the proximal *Parkin* promoter, each of these HREs was individually mutated. The mutation in the first HRE, but not in the second HRE, diminished the induction of promoter activity by hypoxia and CoCl_2_ (Fig. 5D, lower panel), suggesting its crucial role for the HIF-1-activated *Parkin* promoter. We also investigated whether HIF-1α bound to this HRE. We performed a chromatin immunoprecipitation (ChIP) analysis with an anti-HIF-1α antibody and PCR using two sets of primers in Hep3B cells exposed to hypoxia and CoCl_2_ (Fig. 5E, left panel). A single band of ChIP PCR products was detected using primers that amplified regions − 265/ + 17 of the *Parkin* gene with the predicted functional HRE, but not with primers that amplified regions with no HRE (− 1741/− 1510) (Fig. 5E, right panel). These results indicated that HIF-1α bound to the first HRE in hypoxia and enhanced the transcription of *Parkin*.

### The role of Nrf2 and Parkin-Hsc70 on p62-dependent hypoxic cell survival

**Figure 6:**
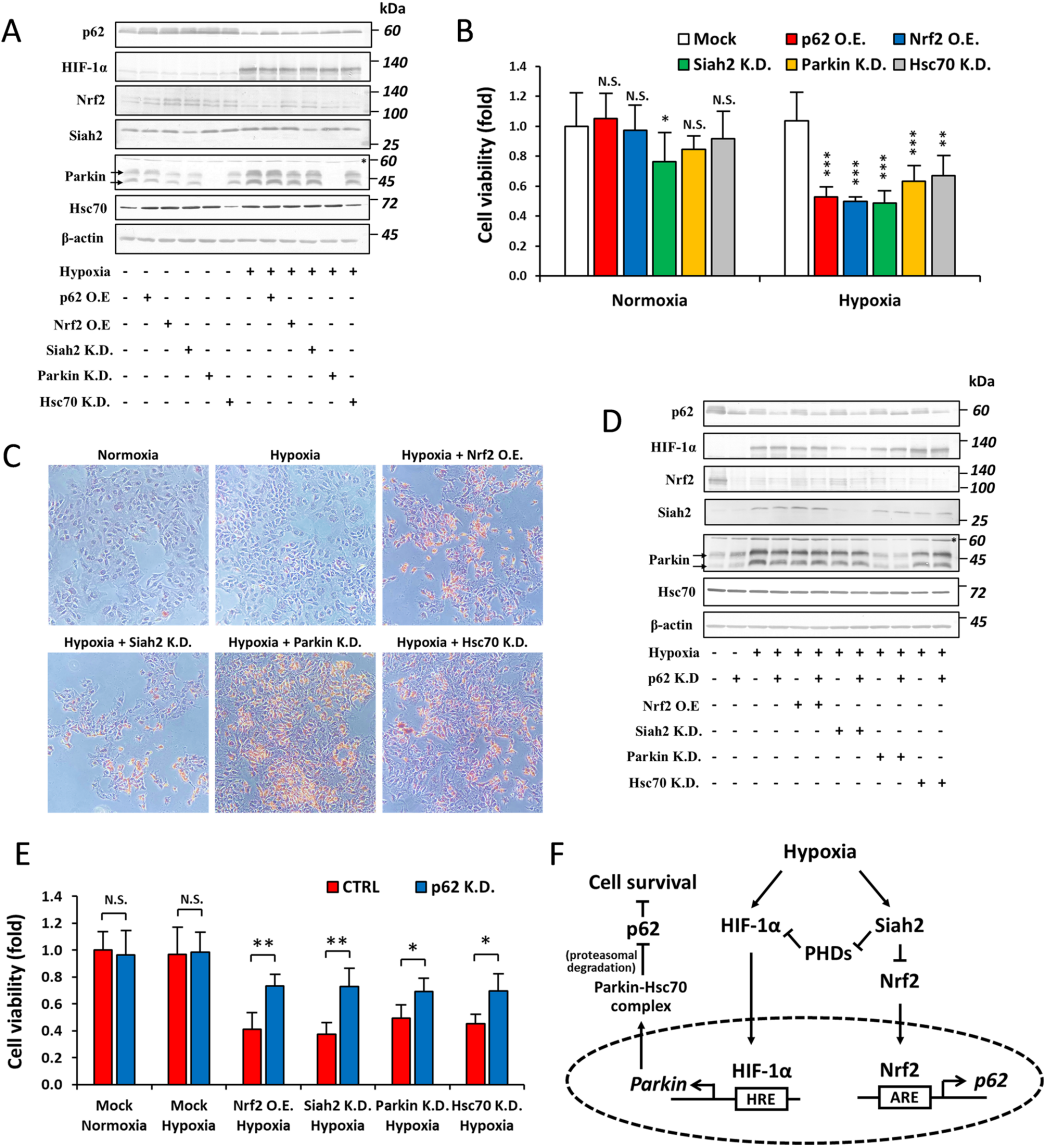
p62 depletion under hypoxia is essential for cell survival*.* (**A**–**C**) Hep3B cells were transfected with pcDNA-p62 (p62 O.E.), pcDNA-Nrf2 (Nrf2 O.E.), sh-RNA Siah2 (Siah2 K.D.), siRNA-Parkin (Parkin K.D.), or siRNA-Hsc70 (Hsc70 K.D.). Cells were then incubated under normoxia or hypoxia (O_2_ = 1%) for 12 h. p62 protein levels (**A**), the viability of cells (**B**), and the number of apoptotic cells (**C**) were then assessed by immunoblotting, the MTT colorimetric assay, and colorimetric TUNEL assay, respectively. (**D** and **E**) Control and p62-knocked down Hep3B cells were transfected with pcDNA-Nrf2, sh-RNA Siah2, siRNA-Parkin, or siRNA-Hsc70 and then incubated under hypoxia (O_2_ = 1%) for 12 h. p62 protein levels (**D**) and the viability of cells (**E**) were assessed by immunoblotting and the MTT colorimetric assay, respectively. (**F**) A working model describing the mechanisms responsible for the depletion of p62 under hypoxia involving transcriptional and post-transcriptional regulation by Nrf2 and Parkin-Hsc70, respectively.

The induction of autophagy and the suppression of p62 under hypoxia have been proposed as the mechanisms promoting tumor cell survival^19,31^. However, the role of a novel mode of the hypoxic regulation of p62, involving Nrf2 and Parkin-Hsc70, in the survival of tumor cells under hypoxia remains unknown. Therefore, we examined the effects of inhibiting the depletion of p62 in hypoxia using strategies involving the overexpression of p62 and Nrf2 as well as the knockdown of Siah2, Parkin, and Hsc70 (Fig. 6A) on the viability of cells. As expected, the prevention of p62 depletion by these strategies reduced the viability of cells under hypoxia (Fig. 6B). Similar phenomena were observed in the examination of apoptosis by the terminal deoxynucleotidyl-transferase-mediated dUTP nick end-labeling (TUNEL) assay (Fig. 6C). To further substantiate the involvement of Nrf2 and Parkin-Hsc70 in hypoxic survival being mediated by p62, we knocked down p62 in Nrf2-overexpressing, Parkin-knocked down, and Hsc70-knocked down Hep3B cells (Fig. 6D). We demonstrated that the viability of cells was recovered by the knockdown of p62 in all of the cells tested (Fig. 6E), confirming the involvement of Nrf2 and Parkin-Hsc70 in hypoxic cell survival in a p62-dependent manner. These results suggest the therapeutic potential of modulating the Nrf2/Parkin-Hsc70 and p62 pathways for the treatment of hypoxic solid tumors.

## Discussion

Hypoxia is a unique microenvironment in solid tumors, including liver cancer, and correlates with a poor prognosis due to its involvement in governing resistance to chemo- and radiotherapy. Targeting pathway(s) involved in cellular responses to hypoxia have been proposed as one strategy to eradicate solid tumors. Under hypoxia, the expression of the autophagy receptor p62 is down-regulated; however, the underlying mechanisms and biological significance of this phenomenon remain unclear. We herein obtained novel results on the mechanisms responsible for the depletion of p62 under hypoxia in the hepatoma cell line Hep3B. Hypoxia activates two arms of the p62 regulatory pathway. HIF-1α and Nrf2 are activated and inhibited under low O_2_ availability, respectively. In one arm, HIF-1α activates the transcription of *Parkin*, which, in turn, forms a complex with Hsc70 to trigger the proteasomal degradation of p62. In the other arm, the Siah2-dependent inhibition of Nrf2 under hypoxia reduces the transcription of *p62*. The depletion of p62 may be used by hypoxic cancer cells as a signaling mechanism to elicit protection and evade apoptosis. Interfering with either arm of this pathway decreased cell survival (Fig. 6F).

The effects of hypoxia on the induction of autophagy have been well-documented in various cellular settings, and this phenomenon is a critical survival mechanism for hypoxic cells via the recycling of cellular components^40–42^. Previous studies showed that the hypoxic clearance of p62 occurred at the post-translational level via autophagy, and the mRNA expression of *p62* was not affected by hypoxia^19,31^. In the present study, we observed a decrease in *p62* mRNA levels in Hep3B cells after 6, 8, and 12 h of hypoxia and in cells treated with 2,2′-dipyridyl and CoCl_2_ for 12 h under normoxia. The present results were supported by Xu et al., who found that hypoxia in rats depleted the hippocampal mRNA expression of *p62*^34^. However, the mechanisms by which hypoxia down-regulates the expression of *p62* at the transcriptional level remain unknown. Various hypoxia-induced cellular response pathways are regulated by HIFs. The present results suggest that HIF-1α and HIF-2α were partly involved in the down-regulation of p62 protein expression, whereas reductions in *p62* mRNA levels were entirely independent of HIFs. We herein revealed that Nrf2 regulated hypoxia-induced decreases in *p62* levels. Nrf2 is considered to regulate the expression of *p62* in response to electrophiles, reactive oxygen species, and nitric oxide, and, as a result of the positive feedback loop, directly binds to ARE in the promoter of the *p62* gene^18,43^. We also demonstrated that Nrf2 regulated not only the inducible expression of *p62*, but also its basal expression, and also down-regulated its expression under hypoxia. Mechanistically, we previously reported that hypoxia induced the phosphorylation of Nrf2 by protein kinase C, thereby inhibiting the Keap1-dependent degradation of Nrf2, and simultaneously stabilized E3 ligase Siah2, which subjected Nrf2 to proteasomal degradation^37^. The prevention of hypoxia-induced decreases in Nrf2 by its overexpression or the knockdown of Siah2 restored reductions in p62 levels. We proved that Nrf2 activity on the previously identified ARE located at − 1258/− 1246 bp in the *p62* promoter^18^ was responsible for this mechanism. To the best of our knowledge, the present study provides the first evidence of the mechanistic down-regulation of *p62* mRNA expression under hypoxia involving Nrf2.

In other cell types, p62 is regulated at the post-transcriptional level^19,31^; therefore, we investigated whether the regulation of p62 at the transcriptional level was the sole mechanism responsible for the hypoxia-mediated suppression of p62. The activation of autophagy under hypoxia in various experimental settings was previously reported to be responsible for the depletion of p62^19,31,44^. In the present study, autophagy inhibitors induced a more robust increase in p62 than proteasome inhibitors under normoxia. However, autophagy inhibitors had a weaker effect on the depletion of p62 by hypoxia than a proteasome inhibitor. A previous study postulated that the regulation of p62 protein stability shifted between proteasomal and autophagic degradation depending on the stress type and severity, which may explain the discrepancy between our results and other findings. For example, mild stress, such as hypoxia, promotes the proteasomal degradation of p62, whereas extreme stress, such as 6-hydroxydopamine, triggers autophagic degradation^22^. In the present study, we demonstrated that the E3 ubiquitin ligase Parkin, a crucial factor in mitophagy and the pathogenesis of Parkinson’s disease, played a critical role in the degradation of p62 under hypoxia, but not normoxia. The role of Parkin on the proteasomal degradation of p62 under hypoxia was previously reported^22^; however, the mechanisms by which hypoxia activates the Parkin-dependent degradation of p62 remain unknown. To clarify the hypoxia-specific regulation of p62 by Parkin, we performed LC–MS and identified Hsc70 as a factor with enhanced binding to Parkin under hypoxia. Parkin and Hsc70 both bind to p62 and are required for hypoxia-induced p62 ubiquitination and degradation. We suggest that Parkin and Hsc70 form a complex that recognizes and targets p62 for proteasomal degradation under hypoxia. Similar to these phenomena, the binding of Hsc70 to Parkin and its role as a recognition domain for Parkin were previously reported for the regulation of Pael receptors (Pael-R), which are involved in endoplasmic reticulum (ER) stress^45,46^. We then demonstrated that hypoxia induced the transactivation of *Parkin* in a HIF-1α-dependent manner via its direct binding to the HRE located − 107 to − 103 bp upstream of the *Parkin* transcription start site. We did not detect any changes in the expression of Hsc70 under hypoxia. These results indicate that Parkin levels are a rate-limiting factor in the proteasomal degradation of p62, and the activation of *Parkin* transcription by HIF-1α enables the post-transcriptional regulation of p62 under hypoxia via the ubiquitin–proteasome system (UPS).

The cellular content of p62 has been used as a marker to study autophagic flux because p62 itself is degraded by autophagy^47^. Under hypoxia, Pursiheimo et al.^19^ and Hu et al.^31^ demonstrated that hypoxia-activated autophagy was responsible for the depletion of p62 in HeLa cells and glioblastoma cell lines, respectively, independent of the UPS and transcriptional regulation. In SHSY5Y cells, Song and colleagues showed the contribution of the UPS involving Parkin to the hypoxic depletion of p62^22^. The participation of *p62* transcriptional regulation was reported by other independent studies on HNSCC^33^ and rat hippocampal tissue^34^. In the present study, we also demonstrated the combination of both the transcriptional and post-translational regulation of p62 in hepatoma cells. Based on evidence for the mechanistic regulation of p62 under hypoxia being highly dependent on the cell type, we speculated that the distinct expression patterns of Nrf2, Siah2, Parkin, and Hsc70 between cell types may contribute to these variations. We then searched the cell line transcriptome online database, The Human Protein Atlas (http://www.proteinatlas.org)^48^, for evidence of these possible variations. HeLa and U87MG cells both express low levels of Nrf2, Siah2, and Parkin, which explains the significant role of the autophagy pathway in the degradation of p62. The expression of Nrf2 and Siah2 was low in SHSY5Y cells, whereas that of Parkin was high, underlying the dependency of the Parkin-dependent proteasomal degradation of p62. The regulation of p62 at the transcriptional level in HNSCC cells and OE19 cells may be due to high expression levels of Nrf2 and Siah2, in contrast to low levels of Parkin despite high levels of Hsc70. In comparisons with other cells, Hep3B cells in the present study expressed higher levels of Nrf2, Siah2, and Parkin, which allowed for an efficient and rapid response to hypoxia at both the transcriptional and post-translational (particularly the UPS) levels (Supplementary Fig. 1). Further studies to provide concrete proof for the cell type-specific mechanisms responsible for the regulation of p62 under hypoxia will be of interest. Regardless of these varying mechanisms, the net output of p62 depletion by hypoxia is conserved and independent of the cell type.

Decreased p62 levels under hypoxia in the Nrf2/Siah2- and Parkin/Hsc70-dependent pathways are of interest because the suppression of p62 is critical for cancer cell survival under hypoxia and, thus, may serve as a novel target for solid tumors. Mechanistically, p62 sequesters and negatively regulates extracellular signal-regulated kinase (ERK) under basal conditions^24^. Jiao and colleagues showed that the activation of ERK reduced O_2_ consumption, decreased CO_2_ release, and lowered energy expenditure^49^; therefore, the suppression of p62 and subsequent activation of ERK may be critical steps in the response to hypoxia. Pursiheimo and colleagues reported that p62 prevented hypoxia-induced ERK phosphorylation^19^. Another independent study showed a reduced mitochondrial membrane potential and decreased O_2_ consumption in *p62* KO mice^50^. Other studies demonstrated that p62 directed metabolic reprogramming from glycolysis to oxidative phosphorylation during neuronal differentiation^51^, suggesting that the inhibition of p62 under hypoxia shifts metabolism to favor glycolysis over oxidative phosphorylation. In addition to the role of p62 in metabolism, we herein showed that the inhibition of p62 was critical for attenuating apoptosis and improving cell survival under hypoxia. Furthermore, the suppression of p62 under hypoxia was proven to play a role in the migration of HNSCC cells^33^, further highlighting the potential use of targeting the p62 pathway in solid tumor hypoxia.

The hypoxic regulation of p62 levels in hepatoma Hep3B cells involving transcriptional and post-translational mechanisms enables the rapid and efficient inhibition of survival pathways involving p62. The biological significance of the present results is best illustrated by the reduced survival of Nrf2-overexpressing, Siah2-knocked down, Parkin-knocked down, and Hsc7-knocked down cells dependent on p62 under hypoxia. Further studies are needed to establish whether the detrimental effects of p62 on hypoxic tumors is mediated by its role in the Ras/ERK signaling or apoptosis pathways, mainly because we currently lack direct evidence for the mechanistic involvement of p62 in the survival pathway under hypoxia. Collectively, the present results suggest that the p62-dependent survival pathway under hypoxia warrants further study as a novel target for solid tumor therapy. Additionally, the discrepancy between our results and other findings reflects a cell type-specific mode of hypoxic p62 regulation; therefore, the careful consideration of tumor types is required when developing strategies for solid tumor therapies targeting these pathways.

## Methods

### Chemicals

NH_4_Cl, 2,2′-dipyridyl, CoCl_2_, Dulbecco’s modified Eagle’s medium (DMEM), ScreenFect™ A, an anti-DYKDDDDK (FLAG) antibody, and an anti-Myc tag monoclonal antibody were purchased from Wako Pure Chemical Industries, Ltd. (Osaka, Japan). Penicillin–streptomycin solution, fetal bovine serum (FBS), geneticin (G418), CHX, bafilomycin A1, and 3-(4,5-dimethyl-2-thiazolyl)-2,5-diphenyl-2H-tetrazolium bromide (MTT) were obtained from Sigma Chemical Co. (St. Louis, MO, USA). MG132 (Z-Leu-Leu-Leu-CHO) was purchased from the Peptide Institute (Osaka, Japan). Isogen was from Nippon Gene (Toyama, Japan), Revert Aid™ M-MuLV Reverse Transcriptase from MBI Fermentas (Vilnius, Lithuania), Go Taq polymerase from Promega (Madison, WI), and KOD Fx Neo from Toyobo (Tokyo, Japan). The DNA Ligation Kit was obtained from Takara Bio Inc. (Shiga, Japan). 4′,6-Diamidino-2-phenylindole (DAPI) was purchased from Dojindo (Kumamoto, Japan). Anti-Parkin and anti-Hsc70 antibodies were from ProteinTech (Rosemont, IL, USA). An anti-ubiquitin antibody (clone FK2) was from StressMarq Bioscience. Alexa Fluor® 594-conjugated goat anti-mouse IgG and Alexa Fluor® 647-conjugated goat anti-rabbit IgG were obtained from Abcam (Carlsbad, CA, USA).

### Cell culture, transfection, and treatment

Human hepatoma cell line Hep3B was obtained from the Cell Resource Center for Biomedical Research at the Institute of Development, Aging and Cancer of Tohoku University (Sendai, Japan). Hep3B cells were cultured in DMEM high glucose containing 10% (v/v) FBS, penicillin (100 units/mL), and streptomycin (100 µg/mL). Cells were maintained at 37 °C in 5% CO_2_ and 95% air (normoxia) or 1% O_2_ (hypoxia) in the multi-gas incubator, APM-30D (Astec, Shizuoka, Japan). Cells were cultured in the presence or absence of MG132 (5 µM, 12 h unless otherwise indicated), NH_4_Cl (10 mM, 12 h unless otherwise indicated), bafilomycin (10 nM, 12 h unless otherwise indicated), and CHX (10 μM, 12 h unless otherwise indicated). The transfection of the indicated constructs was performed using the calcium phosphate method.

### Plasmid constructs

Human Nrf2 cDNA (GenBank™ accession number NM_006164.5) was isolated with primers 1 and 2 (Table 1), digested by *BamH*I and *Xba*I, and then inserted into the pcDNA3.1( +) vector (Invitrogen). Human p62 (NM_003900.5) and Parkin (NM_004562.3) cDNAs were amplified by PCR with primer sets 3 and 4, and 5 and 6, respectively. Amplified p62 and Parkin were then digested with *EcoR*I and *Xba*I, and *BamH*I and *EcoR*I, respectively, and inserted into the 3 × FLAG-pcDNA4 and pcDNA3.1( +) vectors (Invitrogen). Regarding the Parkin-Venus construct, the Venus sequence was inserted into pcDNA3.1( +) with *Not*I, and the cDNA of Parkin without the stop codon was amplified with primer sets 5 and 7 and inserted with *BamH*I and *EcoR*I. In the Myc-Hsc70 construct, the myc-tag sequence was inserted into pcDNA3.1( +) with *Nhe*I and *Kpn*I, and the full-length cDNA of human Hsc70 (NM_006597.6) was isolated with primers 8 and 9 and then inserted with *BamH*I and *Xba*I. In the luciferase reporter assay, genomic DNA was isolated from Hep3B cells, and the sequence upstream of the transcriptional start site of human *p62*, − 1475 to + 46, was amplified using primer sets 10 and 11, digested with *Nhe*I and *Hind*III, and then ligated into the pGL3-promoter vector (Promega). ARE mutant *p62* promoter cDNA was obtained by two rounds of PCR. In the first round of PCR, nucleotide fragments 1 and 2 were amplified with primers 10 and 13, and 11 and 12, respectively. The second round of PCR was performed with fragments 1 and 2 as templates and primers 10 and 11. The upstream region of human *Parkin*, − 360 to + 100, was amplified using primers 14 and 15, digested with *Kpn*I and *Nhe*III, and then ligated into the pGL3-promoter vector. HRE mutant *Parkin* promoter cDNA was obtained by two rounds of PCR. In the first round of PCR, nucleotide fragment 1 was amplified with primers 14 and 17 for HRE mutant 1, and primers 14 and 19 for HRE mutant 2, respectively. Nucleotide fragment 2 was amplified with primers 15 and 16 for HRE mutant 1, and primers 15 and 18 for HRE mutant 2. The second round of PCR was performed with fragments 1 and 2 as templates and primers 14 and 15 for HRE mutant 1 and 2 constructs.

**Table 1:** Primers used for plasmid constructs.

No	Sequences	Descriptions
1	5′- AAGGATCCATC**ATG**ATGGACTTGGAGCT -3′	Fw; 1–17 of human *Nrf2* cDNA; underline, *BamH*I site; bold, start codon
2	5′- TTTCTAGA**CTA**GTTTTTCTTAACATC -3′	Rv; 1801–1818 of human *Nrf2* cDNA; underline, *Xba*I site; bold, stop codon
3	5′- AAGAATTCT**ATG**GCGTCGCTCACCGTGAAGGC -3′	Fw; 1–23 of human *p62* cDNA; underline, *EcoR*I site; bold, start codon
4	5′- TTTCTAGA**TCA**CAACGGCGGGGGATGCTTTG -3′	Rv; 1301–1323 of human *p62* cDNA; underline, *Xba*I site; bold, stop codon
5	5′- AAGGATCC**ATG**ATAGTGTTTGTCAGGTT -3′	Fw; 1–20 of human *Parkin* cDNA; underline, *BamH*I site; bold, start codon
6	5′- TTGAATTC**CTA**CACGTCGAACCAGTGGT -3′	Rv; 1379–1398 of human *Parkin* cDNA; underline, *EcoR*I site; bold, stop codon
7	5′- TTGAATTCAACACGTCGAACCAGTGGTCCC -3′	Rv; 1376–1395 of human *Parkin* cDNA; underline, *EcoR*I site
8	5′- ATTGGATCCGCAACC**ATG**TCCAAGGGAC -3′	Fw; 1–13 of human *Hsc70* cDNA; underline, *BamH*I site; bold, start codon
9	5′- TTTCTAGA**TTA**ATCAACCTCT -3′	Rv; 1929–1941 of human *Hsc70* cDNA; underline, *Xba*I site; bold, stop codon
10	5′- ATTGCTAGCAAACTTCCCGCGATCCCAAACGGG -3′	Fw; upstream of *p62* − 1475 to − 1452; underline, *Nhe*I site
11	5′- TTTAAGCTTCGCGGCTTTTGTAGCGAACGCGGA -3′	Rv; upstream of *p62* + 23 to + 46; underline, *Hind*III site
12	5′- TGGGCTGC**A**G**TA**TCA**TA**CTTGGCCAGCACC -3′	Fw; upstream of *p62* − 1263 to − 1234; underline, ARE site; bold, mutant nucleotides
13	5′- GGTGCTGGCCAAG**TA**TGA**TA**C**T**GCAGCCCA -3′	Rv; upstream of *p62* − 1263 to − 1234; underline, ARE site; bold, mutant nucleotides
14	5′- TATGGTACCAGAAATTCAACCCCTCGTAG -3′	Fw; upstream of *Parkin* − 360 to − 341; underline, *Kpn*I site
15	5′- ATTGCTAGCATGGTCACTGGGTAGGTGGC -3′	Rv; upstream of *Parkin* + 81 to + 100; underline, *Nhe*I site
16	5′- GCAGGAG**ATATA**AGGAGAAA -3′	Fw; upstream of *p62* − 113 to − 95; bold, mutant HRE_1
17	5′- TTTCTCCT**TATAT**CTCCTGC -3′	Rv; upstream of *p62* − 113 to − 95; bold, mutant HRE_1
18	5′- TCCGC**ATATA**CGCATTCCTA -3′	Fw; upstream of *p62* − 49 to − 30; bold, mutant HRE_2
19	5′- TAGGAATGCG**TATAT**GCGGA -3′	Rv; upstream of *p62* − 49 to − 30; bold, mutant HRE_2

### Knockdown experiments

To knockdown endogenous HIF-1α, Nrf2, parkin, and Hsc70, cells were transfected with siRNA against HIF-1α (Cat. No. SI02664053), Nrf2 (SI03246950), Parkin (SI04246550), and Hsc70 (SI02661477) purchased from Qiagen (Hilden, Germany). The knockdown of HIF-2α was performed by siRNA against HIF-2α (Cat. No. AM16708) purchased from Life Technologies (Tokyo, Japan). All siRNAs were transfected using ScreenFect™ A Transfection Reagent (Wako, Osaka, Japan) according to the manufacturer’s instructions. AllStars Negative Control (Cat. No. SI03650318, Qiagen) was used as a control in RNA interference experiments. Regarding the knockdown of Siah2 using shRNA, the target sequence of 5′-ACCCGGAGTGCTTATCTTAAA-3′ was inserted into the pBAsi-hU6 Neo Vector (Takara Bio Inc., Shiga, Japan) according to a previously described procedure^37^. The control for the shRNA experiment was the sequence of 5′-CTCGAGTACAACTATAACTCA-3′ against GFP. Transfectants of shRNA were selected using G418.

### Immunocytochemistry and immunoblotting

Hep3B cells were grown on 3.5-cm glass-bottomed dishes, transfected with pcDNA-Parkin-Venus, and incubated under hypoxia (O_2_ = 1%, 12 h). Cells were then fixed with 4% paraformaldehyde (Wako), blocked in 0.1% bovine serum albumin in TPBS at 4 °C for 1 h, incubated with anti-p62 and anti-Hsc70 (1:1000 dilution in TPBS) at 4 °C for 1 h, and then washed with TPBS. Cells were incubated with Alexa Fluor® 594-conjugated goat anti-mouse IgG and Alexa Fluor® 647-conjugated goat anti-rabbit IgG, and counterstained with DAPI at 4 °C for 1 h. Images were obtained by confocal microscopy (TCS SP8, Leica Microsystem, Wetzlar, Germany). A line scan analysis of confocal images was performed with the RGB profiler plugin in the NIH Image software ImageJ version 1.53t (www.imagej.net) to illustrate the spatial relationships of fluorescent signals. Anti-p62, anti-HIF-1α, anti-HIF-2α, anti-Nrf2, anti-Siah2, anti-Parkin, anti-Hsc70, anti-Myc, anti-FLAG, anti-ubiquitin, and anti-β-actin antibodies were used for immunoblotting. To improve the quantifiability of band intensities, the membranes were cut around the expected molecular size of the protein of interest and loading control (β-actin) prior to hybridization with antibodies. Band intensities were quantified using the NIH Image software ImageJ version 1.53t. The raw uncropped membrane images can be found in Supplementary Fig. 2 to 7.

### Preparation of antibodies

Antibodies against Nrf2, Keap1, HIF-1α, and β-actin were prepared as previously described^37,52^. Antibodies against human HIF-2α, Siah2, and p62 were prepared as follows. HIF-2α (GenBank accession number NM_001430) cDNA (1-741st nucleotide) was isolated using the primer sets 5′-AAGTCGACGACAATGACAGCTGACAAGG-3′ (forward; underlined, the *Sal*I site; double-underlined, the start codon) and 5′-AAGGTACCGCGGCTCAGGAAGGTCTTGC-3′ (reverse; underlined, the *Kpn*I site). Full-length Siah2 (NM_005067) was isolated using the primer sets 5′-AAGGATCCGCGATGAGCCGCCCGTCCTCCAC-3′ (forward; underlined, the *BamH*I site; double-underlined, the start codon) and 5′-TTAAGCTTGAAAGTCACATCATGGACAA-3′ (reverse; underlined, the *Hind*III site; double-underlined, the stop codon). Full-length p62 (NM_003900) was isolated using the primer sets 5′- AAAGGTACCATGGCGTCGCTCACCGTGAAGGC-3′ (forward; underlined, the *Kpn*I site; double-underlined, the start codon) and 5′- TTTAAGCTTTCACAACGGCGGGGGATGCTTTG-3′ (reverse; underlined, the *Hind*III site; double-underlined, the stop codon). HIF-2α, Siah2, and p62 cDNAs were then ligated into pQE-80L vectors (Qiagen, Hilden, Germany). HIF-2α amino acids 1-247, full-length Siah2, and full-length p62 proteins were expressed in *Escherichia coli* strains DH5α (TOYOBO, Tokyo, Japan), BL21 (Novagen, Madison, WI), or Rosetta 2(DE3) (Sigma Chemical Co., St. Louis, MO) by the addition of isopropyl β-d-1-thiogalactopyranoside. The bacterial lysate was solubilized with 1% Triton X-100, deoxycholic acid sodium salt, or CelLytic™ IB (Sigma Chemical Co., St. Louis, MO) for HIF-2α, Siah2, and p62, respectively; the solution was then centrifuged. The resulting supernatant was purified using nickel–nitrilotriacetic acid Agarose (Qiagen, Hilden, Germany) in accordance with the manufacturer’s instructions. HIF-2α, Siah2, and p62 proteins (60 µg each) were injected into a rabbit with Freund’s complete adjuvant for immunization and incomplete adjuvant as a booster (3 times every 2 weeks), and the antibody was raised against HIF-2α, Siah2, and p62 as previously described^53,54^. The cross-reactivity of these antibodies was confirmed using purified proteins from *E. coli* and HIF-2α, Siah2, and p62 overexpressed in Hep3B cells. All experiments were conducted in accordance with guidelines on the welfare of experimental animals and with the approval of the Ethics Committee on the use of animals at Kwansei Gakuin University.

### Semi-quantitative reverse transcription PCR (RT-PCR)

Total RNA was extracted from cells by Isogen according to the manufacturer’s instructions cDNA was synthesized using Revert Aid™ M-MuLV Reverse Transcriptase and was then amplified with Go Taq polymerase and each primer set (Table 2). PCR products were analyzed using 1% agarose gel electrophoresis, visualized with ethidium bromide staining, and quantified using the NIH Image software ImageJ version 1.53t. The raw uncropped gel images can be found in Supplementary Fig. 8 to 10.

**Table 2:** List of primers used for a gene expression analysis by RT-PCR.

Genes	Forward	Reverse
p62	CTACGACTTGTGTAGCGTCT	CCAACGTTCTTCAGGAAATT
HIF-1α	CCTAACGTGTTATCTGTCGC	GTCAGCTGTGGTAATCCACT
HIF-2α	GGCCCAGCTGGCTCCCACCC	GGATATAGGGTGCCAGTGTC
CA9	GCTTCCAGCTCCCGCCGCTC	CCGGGCCCTCCTCCAGAAAG
Epo	ATGTGGATAAAGCCGTCAGTGG	CTGGAGTGTCCATGGGACAG
Nrf2	GCCATTCACTCTCTGAACTT	GGTGACAAGGGTTGTACCAT
NQO-1	TGATCGTACTGGCTCACTCA	GTCAGTTGAGGTTCTAAGAC
Siah2	CACGAGCTGACCTCGCTCTT	TGCCACTTGCAGGAAGCACC
Parkin	TCACATTGTGCAGAGACCGT	GGACTTCCAGCTGGTGGTGA
β-actin	CAAGAGATGGCCACGGCTGCT	TCCTTCTGCATCCTGTCGGCA

### Cell viability assay

Cell viability was performed using the MTT assay (Sigma Chemical Co., St. Louis, MO, USA) according to the manufacturer’s protocol. In brief, Hep3B cells (5 × 10^4^ cells/well) were cultured in 24-well plates and treated with 100 μl of MTT solution (5 mg/ml in PBS) for 2 h. Culture medium was removed, and the resulting purple formazan was dissolved with 500 μl of isopropanol containing 0.04 N HCl and 0.1% NP40. Absorbance was measured at 590 nm using a microplate reader (PerkinElmer, Waltham, MA). Cell viability was calculated as a relative percentage to the control.

### TUNEL assay

Cells were cultured in 6-well plates containing coverslips, transfected with pcDNA-p62, pcDNA-Nrf2, shRNA-Siah2, siRNA-Parkin, and siRNA-Hsc70, and incubated under hypoxia (O_2_ = 1%, 12 h). After treatment, the coverslips were washed with PBS and fixed in 4% paraformaldehyde at room temperature for 25 min. Apoptotic cells were detected by the TUNEL assay using the DeadEnd™ Colorimetric TUNEL System Kit (Promega) according to the manufacturer’s instructions. Apoptotic cells were visualized and photographed with a digital camera (Moticam 2000, Shimadzu Rika Kikai, Tokyo, Japan) coupled to an inverted microscope (CK30-SLP; Olympus, Tokyo, Japan) under × 10 objective magnification. The number of apoptotic cells was calculated as a percentage of apoptotic nuclei (dark brown nuclei) versus total nuclei, evaluated from 3 experiments.

### Luciferase reporter gene assay

Hep3B cells were seeded on 24-well plates and co-transfected with 0.25 µg of the luciferase reporter gene of interest (pGL3-containing *p62* promoter − 1475/ + 46, either the WT or ARE mutant, and pGL3-containing *Parkin* promoter − 360/ + 100, either the WT or HRE mutants), 12.5 ng of pRL-null, and 0.25 µg of pcDNA-Mock or pcDNA-Nrf2 with the GenePORTER TM2 transfection reagent (Gene Therapy Systems). Two days after transfection, cells were incubated under normoxia or hypoxia (O_2_ = 1%) for 12 h with or without 100 µM CoCl_2_, lysed in 65.2 µl of lysis buffer (Promega), and luciferase activity was assayed with a luminometer (Lumat LB9507; Berthold) using the Dual-Luciferase Reporter Assay System (Promega) as per the manufacturer’s protocol. Firefly luciferase activity was normalized to Renilla luciferase activity.

### ChIP assay

The ChIP assay was performed according to a previously described method with minor modifications^55,56^. Hep3B cells were cultured under normoxia, hypoxia (O_2_ = 1%), or treated with CoCl_2_ (100 µM, normoxia) for 12 h, incubated in PBS containing 1.5% (w/v) formaldehyde at room temperature for 10 min, and 0.125 M of glycine was added to stop the crosslinking reaction. Cells were washed with ice-cold PBS, lysed in ChIP buffer (150 mM NaCl, 50 mM Tris–HCl pH 7.5, 0.5 mM DTT, 5 mM EDTA, 0.5 mM PMSF, 0.5% NP-40, 1% Triton X-100, and 10 mM NaF), and sonicated on ice to shear DNA. After centrifugation, 50 μl of the supernatant was collected as the input. The remnant was immunoprecipitated using protein A-Sepharose beads coated with control IgG from an unimmunized rabbit or the anti-HIF-1α antibody at 4 °C for 45 min. Beads were extensively washed and eluted. Genomic DNA was purified by the phenol–chloroform extraction method followed by RT-PCR using the primer sets 5′- TCACCTGACACACTTCCCAA -3′ (forward) and 5′- CACCCTGGGAAGATCTGAGT -3′ (reverse) to amplify the − 1741/− 1510 fragment, and 5′- CAGGACCTTGGCTAGAGCTG -3′ (forward) and 5′- CTCCTGGGTTAAATCCTCCA -3′ (reverse) to amplify the − 265/ + 17 fragment of the *Parkin* promoter.

### Statistical analysis

All data are presented as the mean ± standard deviation (SD) from triplicate experiments and analyzed with IBM SPSS Statistics for Windows, version 23.0. Statistical comparisons were performed with the Student’s *t*-test or a one-way analysis of variance (ANOVA) with the respective post-hoc tests for multiple comparisons against specified groups as described in the figure legends. Differences were considered to be significant when *p* < 0.05 (*), < 0.01 (**), or < 0.001 (***).

## Supplementary Information

Supplementary Figures.

## Data Availability

The datasets generated and/or analyzed during the present study are available from the corresponding author upon request.
