# Supplementary material for: Nrf2 and Parkin-Hsc70 regulate the expression and protein stability of p62/SQSTM1 under hypoxia
Source: Sci Rep. 2022 Dec 8;12:21265. doi: 10.1038/s41598-022-25784-0 (PMC9731985; doi:10.1038/s41598-022-25784-0)
Supplement: Supplementary file 1 — Supplementary Figures. [file 41598_2022_25784_MOESM1_ESM.pdf]

SUPPLEMENTARY FIGURES

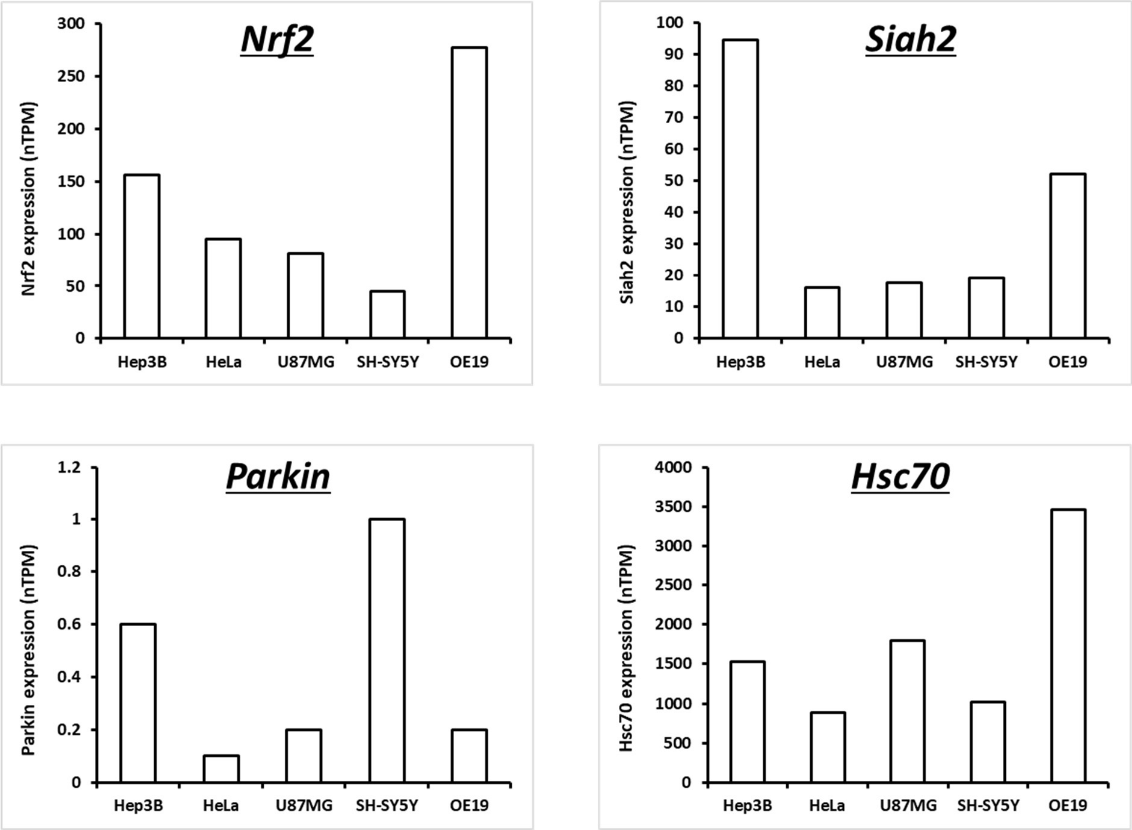

Supplementary Figure 1. A comparison of *Nrf2*, *Siah2*, *Parkin*, and *Hsc70* expression between Hep3B, HeLa, U87MG, SH-SY5Y, and OE19 cell lines. The data were queried from the cell line transcriptome online database, The Human Protein Atlas (www.proteinatlas.org). Data were expressed as normalized transcripts per million (nTPM)

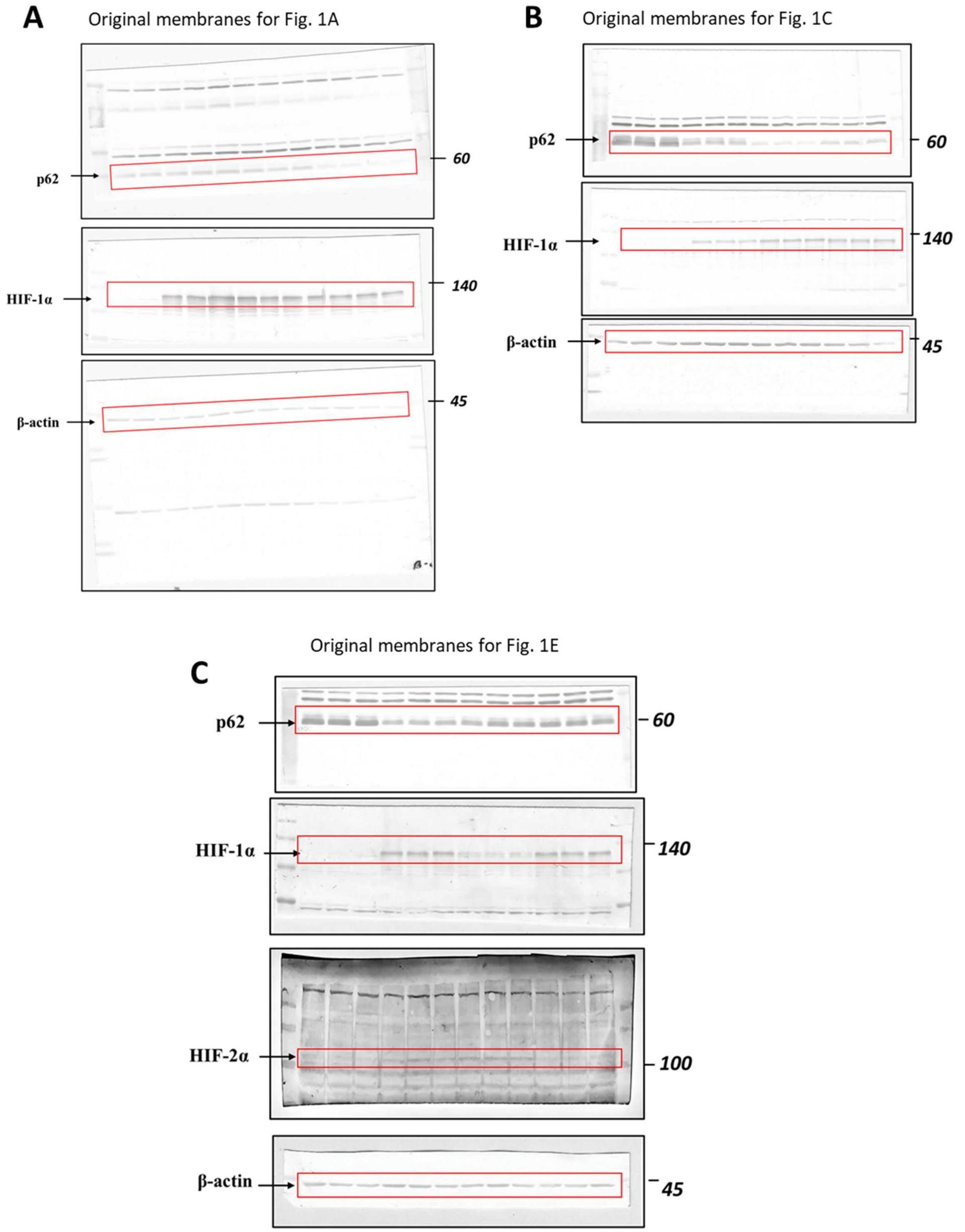

Supplementary Figure 2. Uncropped blot images of the pictures used in Figure 1, show the p62, HIF-1α, HIF-2α, and β-actin bands. The membranes were cut around the expected molecular size of the protein of interest prior to hybridization with antibodies. (A) Original membranes used in Fig. 1A. (B) Original membranes used in Fig. 1C. (C) Original membranes used in Fig. 1E.

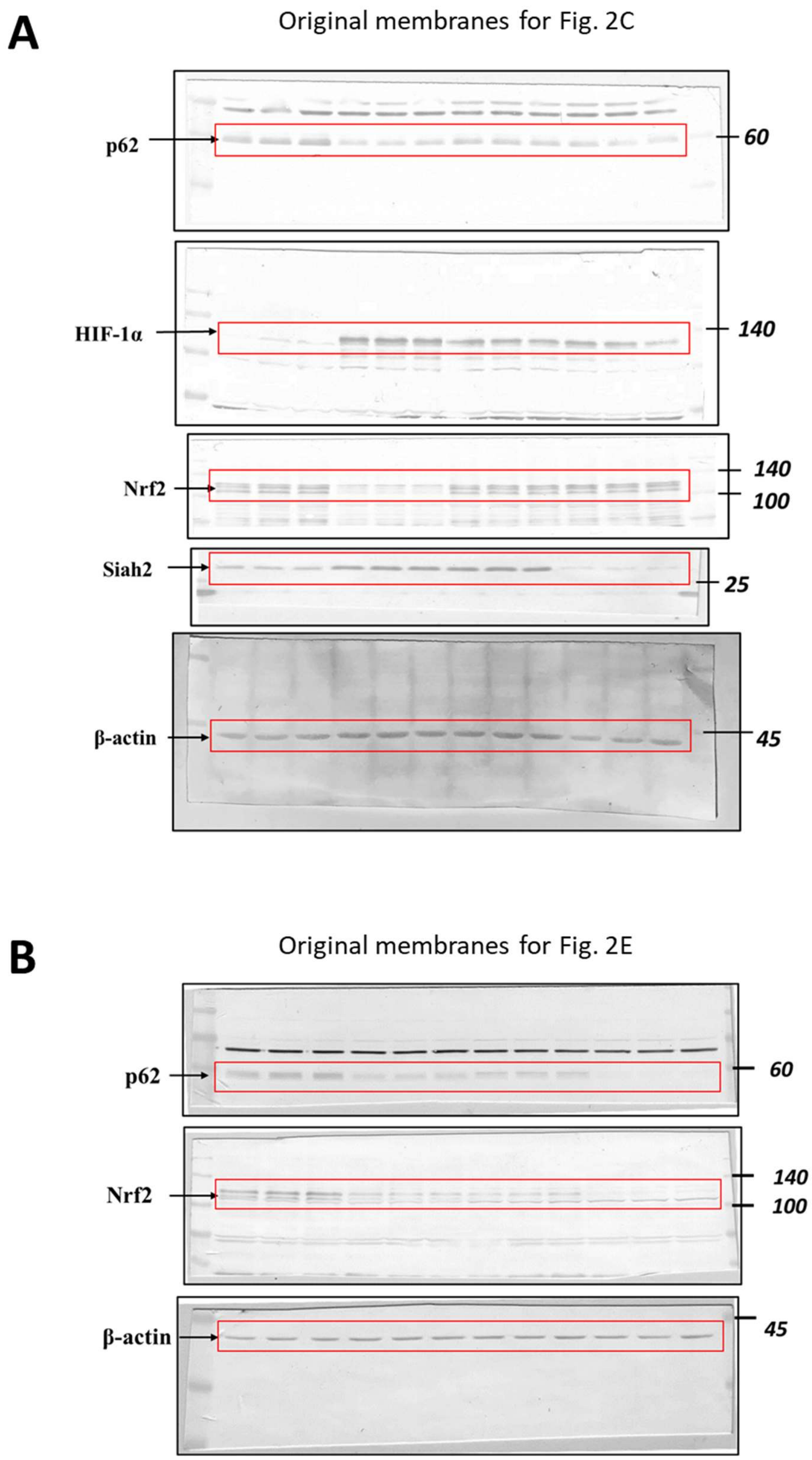

Supplementary Figure 3. Uncropped blot images of the pictures used in Figure 2, show the p62, HIF-1α, Nrf2, Siah2, and β-actin bands. The membranes were cut around the expected molecular size of the protein of interest prior to hybridization with antibodies. (A) Original membranes used in Fig. 2C. (B) Original membranes used in Fig. 2E.

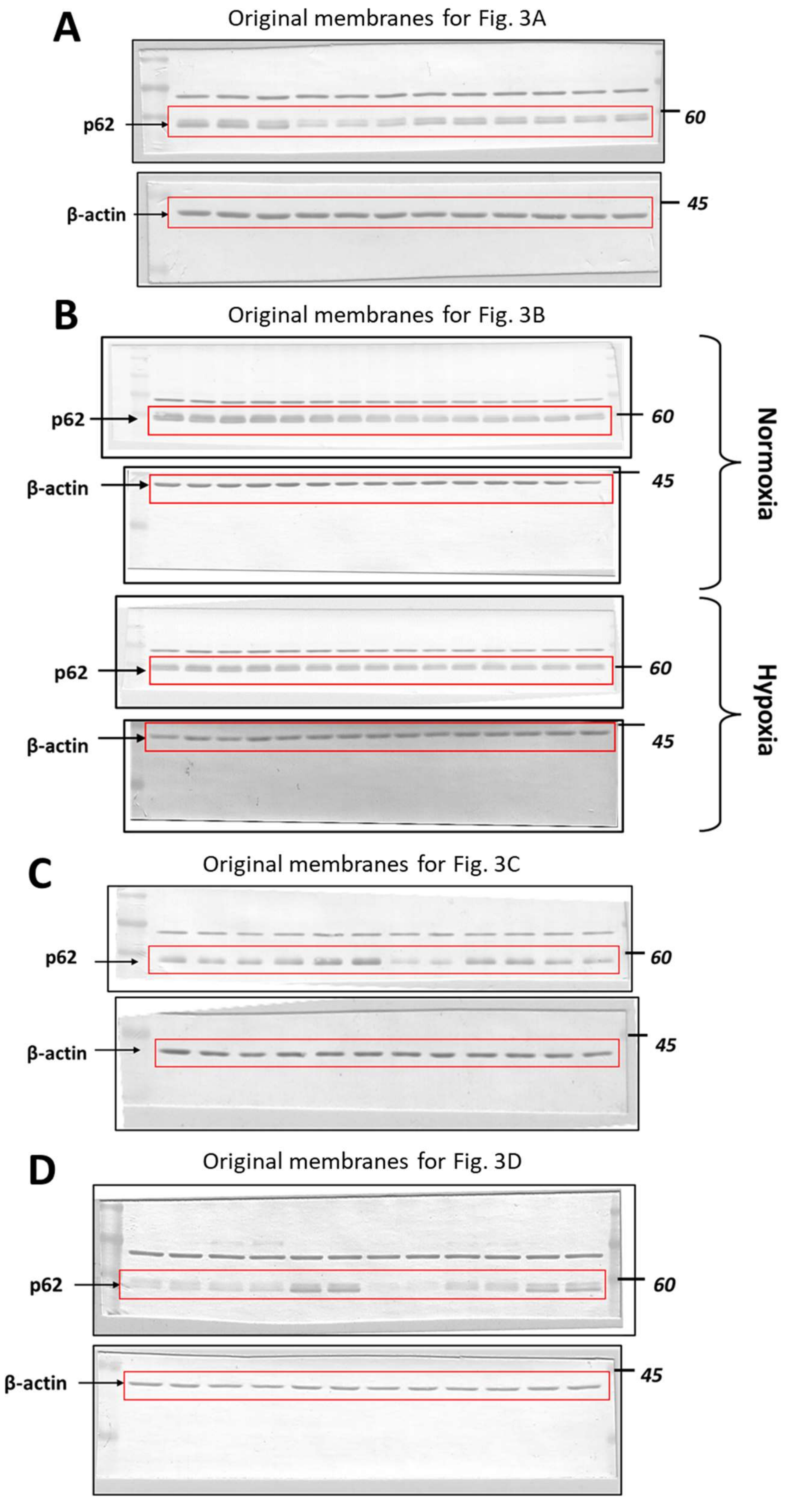

Supplementary Figure 4. Uncropped blot images of the pictures used in Figure 3, show the p62 and β-actin bands. The membranes were cut around the expected molecular size of the protein of interest prior to hybridization with antibodies. (A) Original membranes used in Fig. 3A. (B) Original membranes used in Fig. 3B. (C) Original membranes used in Fig. 3C. (D) Original membranes used in Fig. 3D.

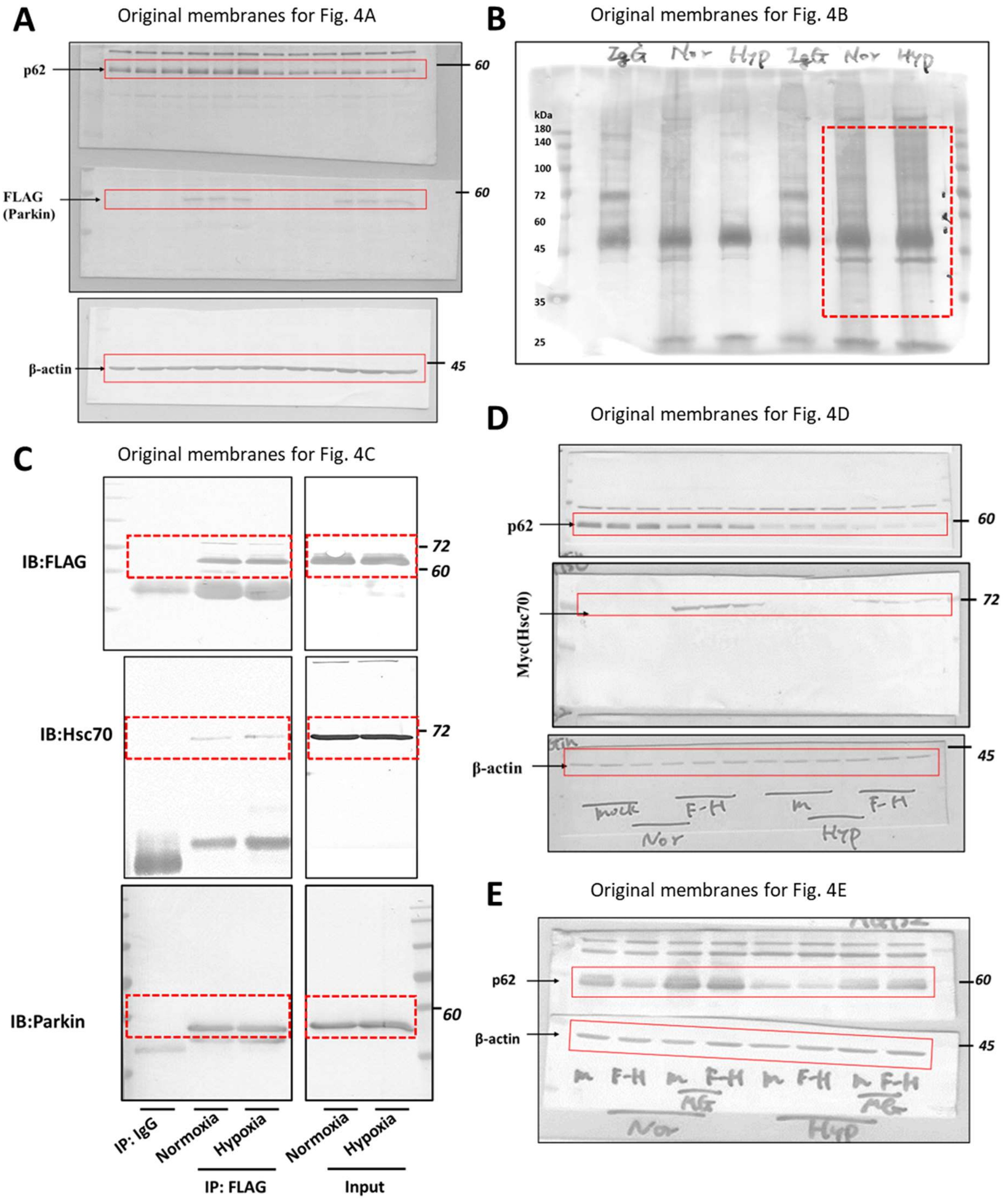

Supplementary Figure 5. Uncropped blot images of the pictures used in Figure 4A-E, showing the p62, FLAG(Parkin), FLAG(p62), Hsc70, Myc(Hsc70), FLAG(Hsc70), Parkin, HIF-1α, Ubiquitin, and β-actin bands. The membranes were cut around the expected molecular size of the protein of interest prior to hybridization with antibodies. (A) Original membranes used in Fig. 4A. (B) Original membranes used in Fig. 4B. (C) Original membranes used in Fig. 4C. (D) Original membranes used in Fig. 4D. (E) Original membranes used in Fig. 4E.

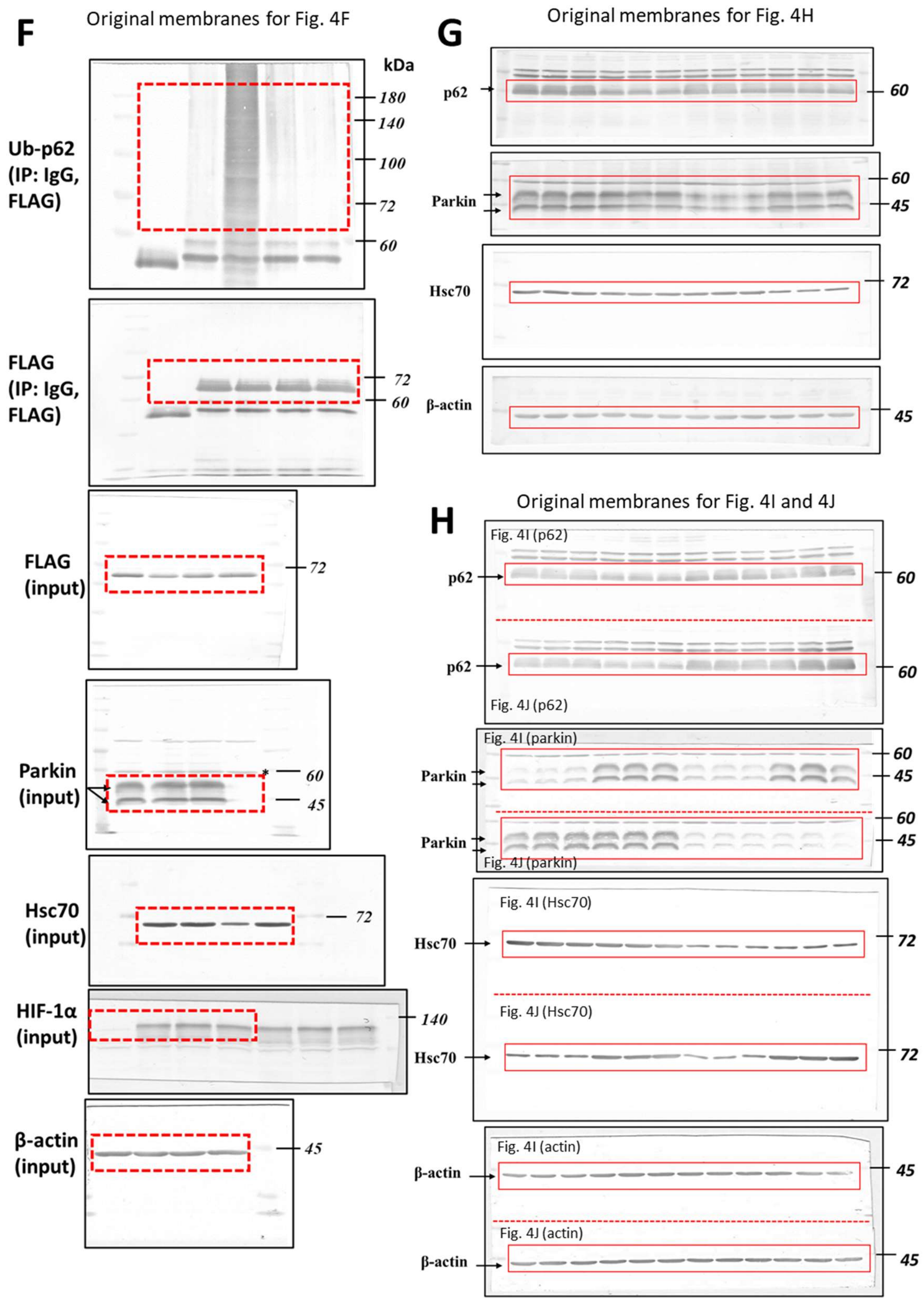

Supplementary Figure 5 (continue). Uncropped blot images of the pictures used in Figure 4F-J, showing the p62, FLAG(Parkin), FLAG(p62), Hsc70, Myc(Hsc70), FLAG(Hsc70), Parkin, HIF-1α, Ubiquitin, and β-actin bands. The membranes were cut around the expected molecular size of the protein of interest prior to hybridization with antibodies. (F) Original membranes used in Fig. 4F. (G) Original membranes used in Fig. 4H. (H) Original membranes used in Fig. 4I. and Fig. 4J.

Original membranes for Fig. 5A

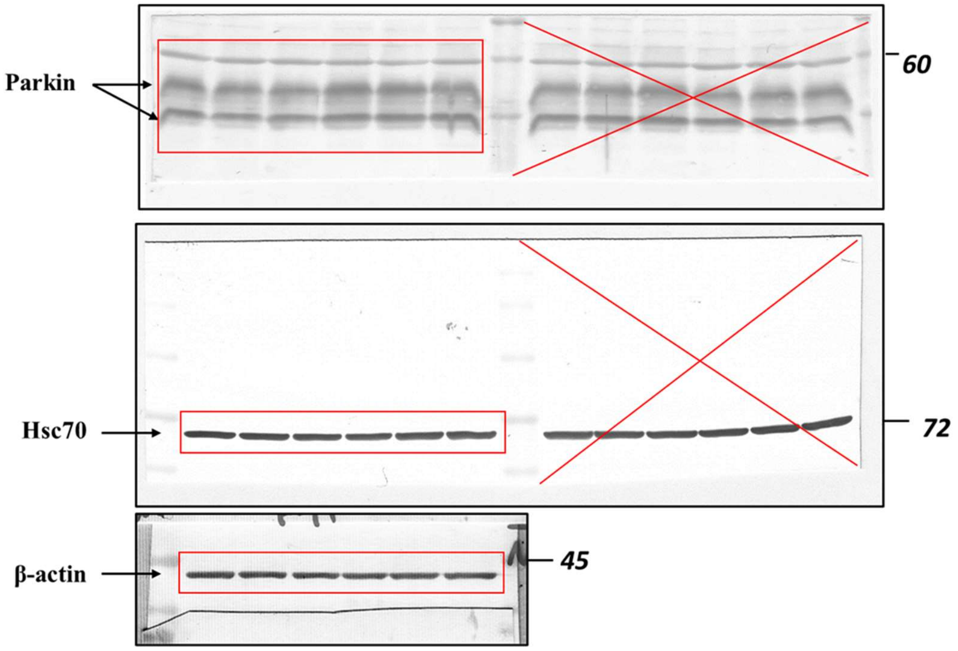

Supplementary Figure 6. Uncropped blot images of the pictures used in Figure 5A, show the Parkin, Hsc70, and β-actin bands. The membranes were cut around the expected molecular size of the protein of interest prior to hybridization with antibodies.

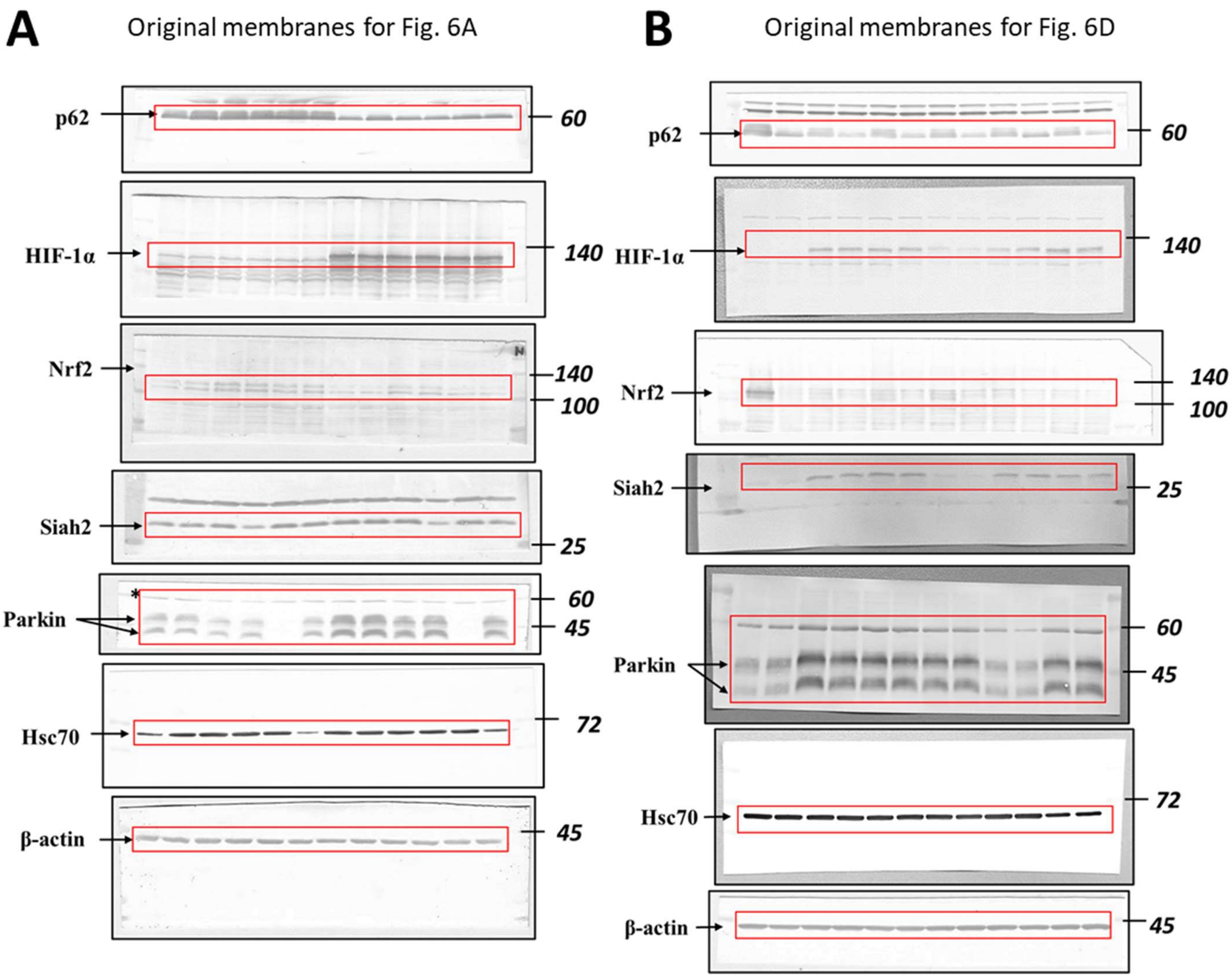

Supplementary Figure 7. Uncropped blot images of the pictures used in Figure 6, show the p62, HIF-1α, Nrf2, SIah2, Parkin, Hsc70, and β-actin bands. The membranes were cut around the expected molecular size of the protein of interest prior to hybridization with antibodies. (A) Original membranes used in Fig. 6A. (B) Original membranes used in Fig. 6D.

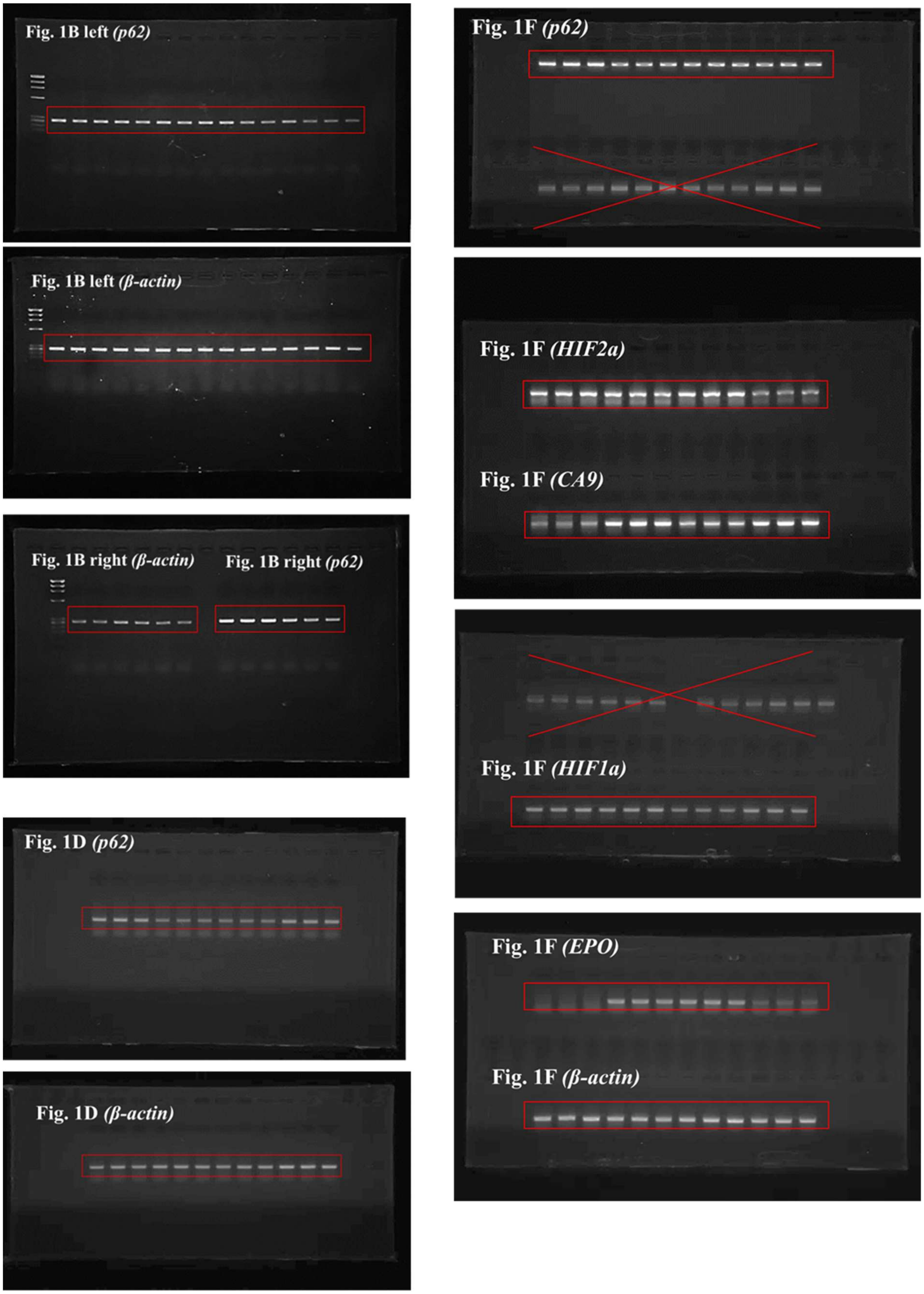

Supplementary Figure 8. Uncropped electrophoresis gels images of the pictures used in Figures 1B, D, and F, show the *p62, HIF-1α, HIF-2α, CA9, Epo, and β-actin* bands.

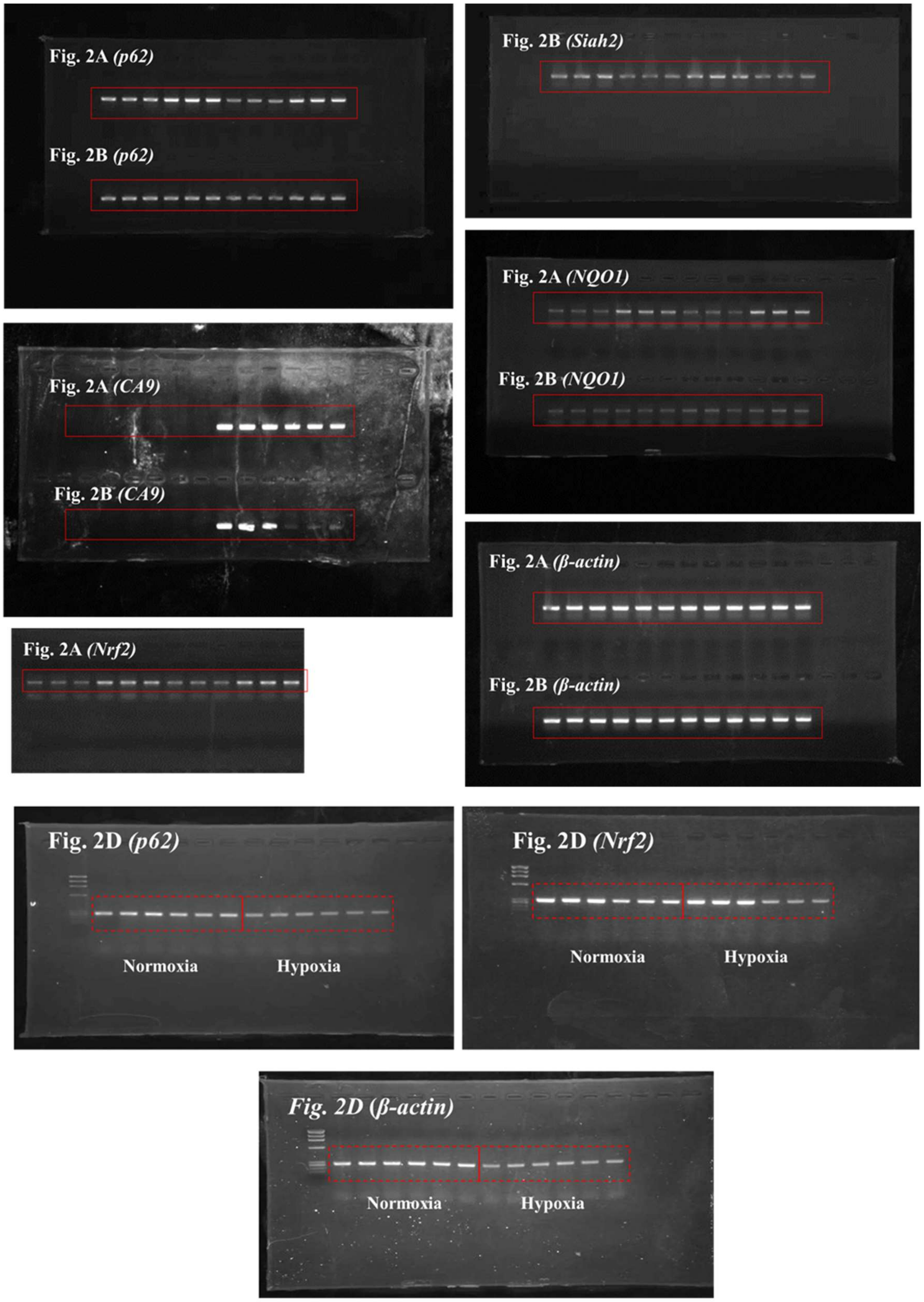

Supplementary Figure 9. Uncropped electrophoresis gels images of the pictures used in Figures 2A, B, and D, show the *p62, CA9, Nrf2, Siah2, NQO1, and β-actin* bands. The PCR results in Fig. 2D (normoxia and hypoxia) are part of one gel and the same experiment. However, we split the picture of Normoxia and Hypoxia in the main manuscript because we analyzed them separately.

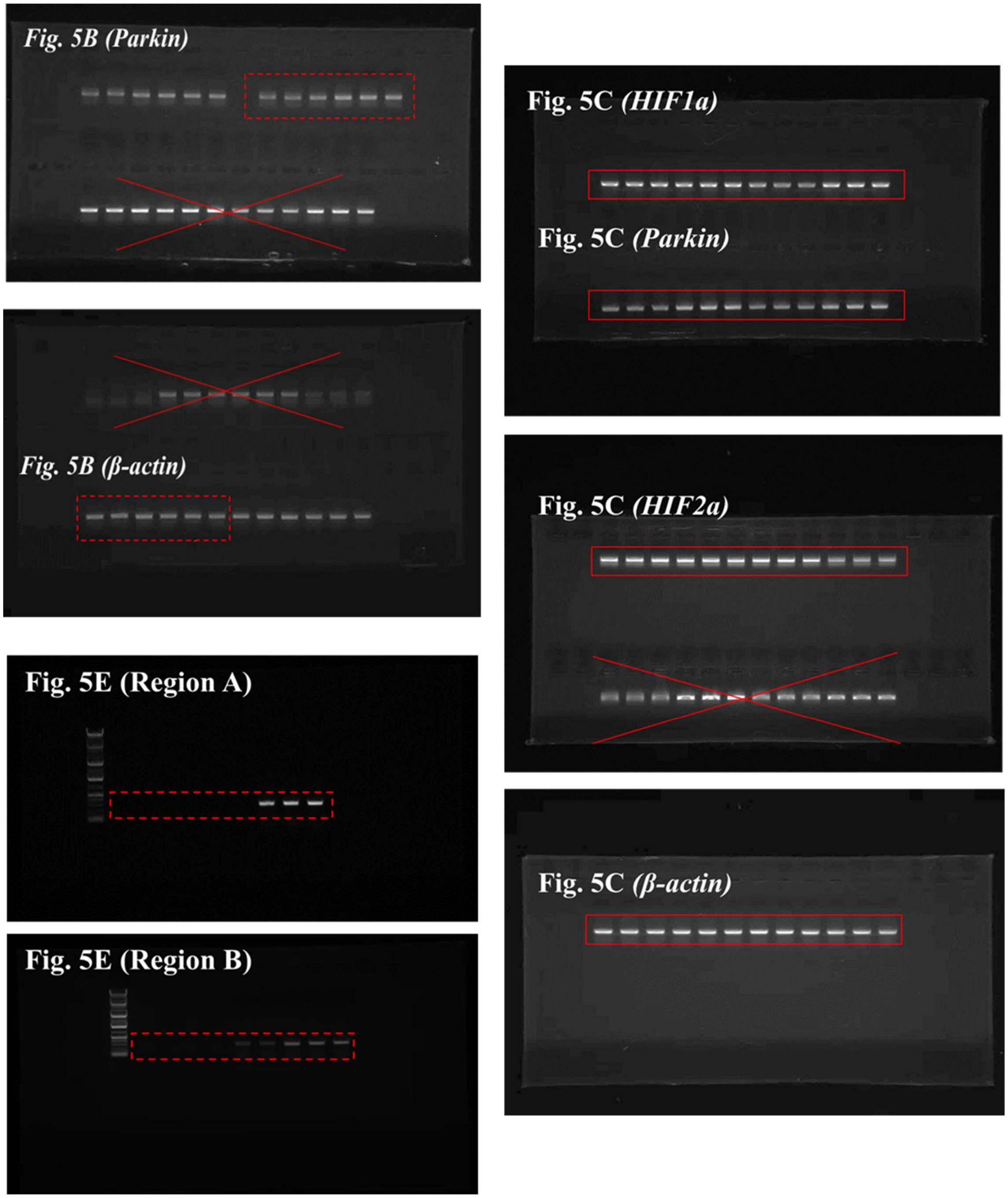

Supplementary Figure 10. Uncropped electrophoresis gels images of the pictures used in Figures 5B, C, and E, show the *Parkin, HIF-1α, HIF-2α,* and *β-actin* bands. The PCR products amplified with the indicated primer sets of the ChIP assay were shown. Note that the gel picture of *β-actin* in Fig. 5B is cropped version and re-used from the *β-actin* of Fig. 1F. We re-used the same gel because the *Parkin* mRNA in Fig. 5B was examined from the same sample as the Fig. 1F, but only for the Normoxia and Hypoxia treatment.
